# Adjuvant dendritic cell-based immunotherapy in melanoma: insights into immune cell dynamics and clinical evidence from a phase II trial

**DOI:** 10.1186/s12967-025-06403-8

**Published:** 2025-04-18

**Authors:** Jenny Bulgarelli, Claudia Piccinini, Emanuela Scarpi, Giorgia Gentili, Laura Renzi, Silvia Carloni, Francesco Limarzi, Elena Pancisi, Anna Maria Granato, Massimiliano Petrini, Francesco De Rosa, Massimo Guidoboni, Dalila Fanelli, Maria Maddalena Tumedei, Marcella Tazzari, Stefano Baravelli, Ilaria Bronico, Pietro Cortesi, Sara Pignatta, Laura Capelli, Valentina Ancarani, Giovanni Foschi, Livia Turci, Francesca Tauceri, Massimo Framarini, Laura Ridolfi

**Affiliations:** 1 Advanced Cellular Therapies and Rare Tumors Unit, IRCCS Istituto Romagnolo per lo Studio dei Tumori (IRST) “Dino Amadori”, 47014 Meldola, Italy; 2 Unit of Biostatistics and Clinical Trials, IRCCS Istituto Romagnolo per lo Studio dei Tumori (IRST) “Dino Amadori”, 47014 Meldola, Italy; 3 Medical Genetics Unit, AUSL Romagna, 47522 Cesena, Italy; 4 Pathology Unit, Morgagni-Pierantoni Hospital, AUSL Romagna, 47121 Forlì, Italy; 5 Department of Oncology, University Hospital of Ferrara, 44124 Ferrara, Italy; 6 Unit of Immunohematology and Transfusion Medicine, Morgagni-Pierantoni Hospital, AUSL Romagna, 47121 Forlì, Italy; 7 Radiotherapy Unit, IRCCS Istituto Romagnolo per lo Studio dei Tumori (IRST) “Dino Amadori”, 47014 Meldola, Italy; 8 Cardio-Oncology Unit, IRCCS Istituto Romagnolo per lo Studio dei Tumori (IRST) “Dino Amadori”, 47014 Meldola, Italy; 9 Bioscience Laboratory, IRCCS Istituto Romagnolo per lo Studio dei Tumori “Dino Amadori” (IRST), 47014 Meldola, Italy; 10 Advanced Oncological Surgery Unit, Morgagni-Pierantoni Hospital, AUSL Romagna, 47121 Forlì, Italy

**Keywords:** Dendritic cell, Immunotherapy, Skin cancer, Immune modulatory, Tumor microenvironment

## Abstract

**Background:**

Dendritic cells (DCs) are the most efficient antigen-presenting cells and play a central role in the immune system, orchestrating immune response against tumors. We previously demonstrated that DC-based vaccination effectively induces anti-tumor immunity, yet at the same time showing a robust safety profile, making this treatment a potential candidate for effective adjuvant immunotherapy. To explore this possibility, we designed a randomized phase II trial (EudraCT no. 2014-005123-27) to provide a complementary autologous DC vaccination to patients (pts) with resected stage III/IV melanoma.

**Methods:**

Overall, a total of 18 eligible pts were included in this study, 10 of whom received 6 monthly DC vaccination cycles combined with IL-2 administration (arm A), and 8 pts were enrolled in the follow-up observational cohort (arm B). A deep immune biomarkers profiling by multiplex immunoassay, human leukocyte antigens (HLA) typing, multiparametric flow cytometry and in situ tumor microenvironment analysis was performed for the entire pts cohort. The immunological response was assessed in vivo by DTH test and ex vivo against selected melanoma-associated antigens applying the IFN-γ ELISPOT assay.

**Results:**

Pts receiving DC vaccination showed a better relapse-free survival compared to the observational cohort (median 6.6 months, 95% CI, 2.3–not reached (nr) (arm A) vs 5.2 months, 95% CI, 2.5–nr (arm B), not significant), with a favorable trends for female pts (median 15.5 months, 95% CI, 2.6–nr (female) vs 3.3, 95% CI, 2.3–nr (male)), pts with less than 60 years (median 22.5 months, 95% CI, 2.6–nr (age < 60) vs 4.7 months, 95% CI, 2.3–nr (age ≥ 60), and pts with wild-type BRAF status (median 22.5 months, 95% CI, 8.6–nr (BRAF wt) vs 3.8 months, 95% CI, 2.3–nr (BRAF mutated). The toxicity profile was favourable, with no severe adverse events and only mild, manageable reactions. Moreover, additional immune response data suggested increased immune modulation in vaccinated patients, which may reflect a shift in immune dynamics.

**Conclusions:**

Our findings support the safety and tolerability of DC vaccination as an adjuvant treatment for melanoma, demonstrating significant immune modulation at both the tumor site and peripherally in relapsed and non-relapsed patients. These results highlight the potential of autologous, personalised DC-based therapies and pave the way for the development of innovative immunotherapy combinations in future treatment strategies.

*Trial registration* ClinicalTrials.gov NCT02718391; EudraCT no. 2014-005123-27.

**Supplementary Information:**

The online version contains supplementary material available at 10.1186/s12967-025-06403-8.

## Background

Patients (pts) with early-stage melanoma are cured by surgical resection. However, for higher-risk disease, there have been limited systemic treatment options to improve outcomes from surgery alone. After the historic use of interferon-alfa in the adjuvant setting for pts with high-risk resected stage III melanoma, the breakthrough of immune checkpoint inhibitors (ICIs) really changed the story and the clinical benefit for pts with both metastatic and, more recently, resected stage IIB-C, III and resected stage IV melanoma (AJCC VIII edition) [1]. Ipilimumab was the first ICI to demonstrate superior recurrence-free survival and (OS) compared to placebo in pts with stage III melanoma in a randomised phase III trial [2, 3]. In 2015, the Food and Drug Administration approved Ipilimumab for the adjuvant treatment of high-risk melanoma pts. However, it has not been widely used due to its toxicity.

Nowadays, there are several therapeutic options for stage III BRAF mutated or wild-type melanoma in the adjuvant setting, but none of these have shown a statistically significant OS benefit (although dabrafenib/trametinib showed an important trend in favour of combination therapy over placebo), and all of these therapies have a toxicity profile that requires careful consideration [4–6]. To date, there is only one small randomised phase II study that showed an OS benefit of ipilimumab + nivolumab versus placebo in resected stage IV melanoma, but we have to take into account the very small sample size of the study and that OS was not the primary endpoint [7].

Promising results have been achieved with cell therapies and combination strategies. Specifically, DCs are crucial in tumor immunotherapy as potent antigen-presenting cells. They uptake, process, and present tumor-associated antigens (TAAs) to activate cytotoxic immunity. Ex vivo generated DCs have been tested in numerous clinical trials, demonstrating that DC-based vaccines are both safe and effective [8]. In a colon cancer study, the vaccination arm achieved a median relapse-free survival (RFS) of 25.26 months compared to just 9.53 months in the observation arm [9]. In particular, adjuvant treatment with DC vaccines could reduce the risk of recurrence in pts with residual occult disease after surgical resection and induce a durable tumor regression in treated pts [10]. Boudewijns et al. demonstrated the ability of DC-based immunotherapy to induce antigen-specific T cells in stage III compared to stage IV melanoma pts [11]. Finally, Bol KF. et al. retrospectively analysed pts with stage III melanoma who received adjuvant naturally circulating DC immunotherapy and showed an OS advantage compared to their matched controls which unfortunately was not confirmed in the subsequent phase 3 trial [12, 13]

Based on our previous encouraging results with an autologous DC vaccine in metastatic melanoma pts, showing an overall clinical benefit in 54.1% of pts with a very favourable toxicity profile [14], and on the rationale described above, we designed a randomised phase 2 trial in high-risk (stage IV or metachronous stage III) radically resected melanoma pts to compare 6 months of DC vaccine adjuvant therapy with the standard of care being followed at the time.

## Methods

Materials and methods are described in detail in the Additional file 1.

### Study design and treatment schedule

**Fig. 1:**
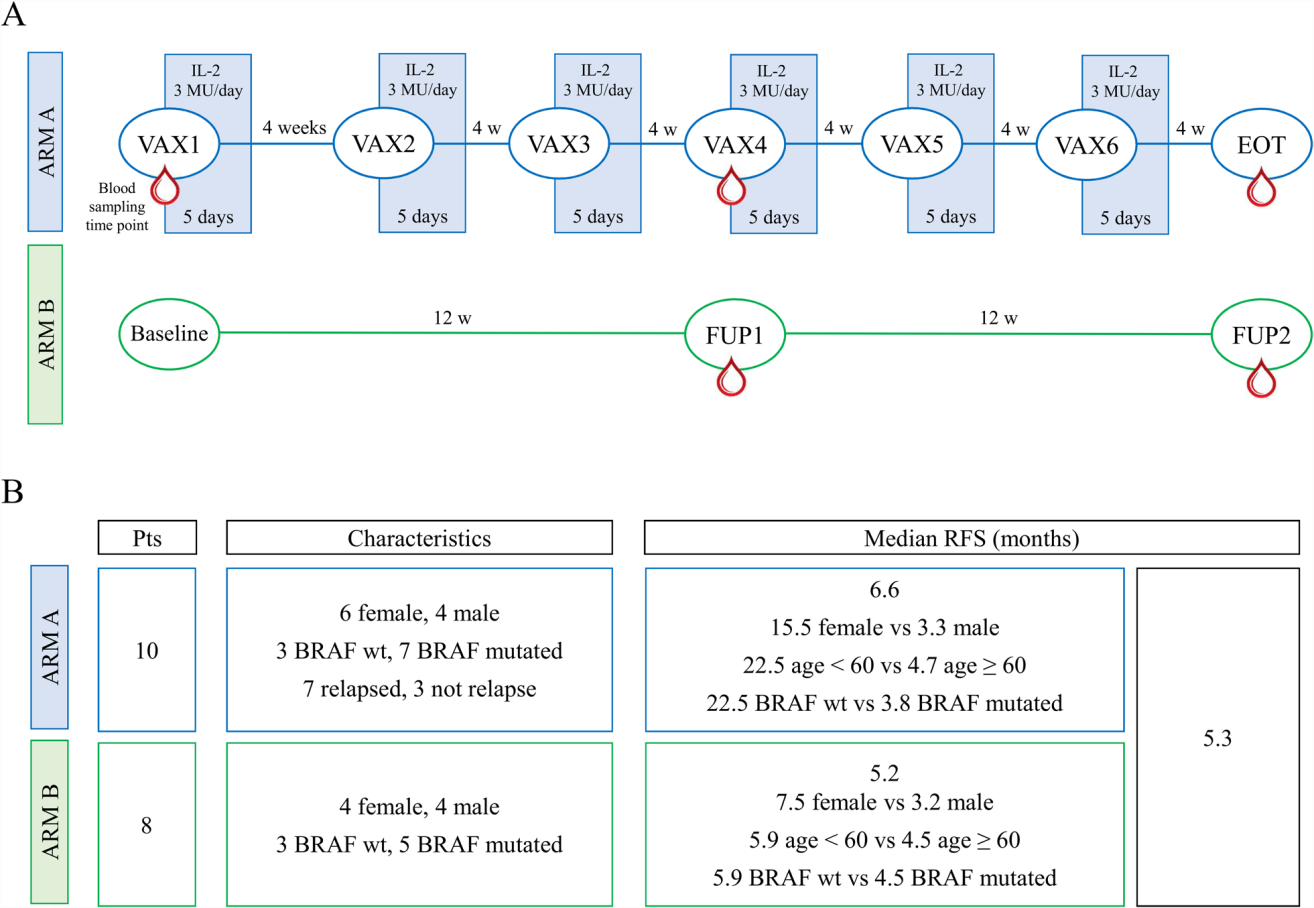
**A** ACDC clinical protocol study design. arm A pts were vaccinated with 6 doses of DC vaccine following total resection, while untreated arm B pts were followed up post-surgery over time. **B** Schematic table in which, for each study arm, the principal baseline pt characteristics and the results of the univariate analysis on RFS (time expressed in months) are summarized

This was a randomized phase II clinical trial (ClinicalTrials.gov NCT02718391; EudraCT no. 2014–005123-27) in completely resected metachronous stage III and stage IV melanoma pts with tissue sample availability. Pts who have undergone previous lines of systemic chemotherapy, immunotherapy or biological therapy for metastatic melanoma were excluded as previously described [15]. The study was approved by the CEIIAV Ethics Committee (approval n° 1231 of 30/07/2015) and was conducted in accordance with the principles laid down in the 1964 Declaration of Helsinki. Written informed consent was obtained from all participants, then pts were randomized in a treatment arm (A) or in a follow-up/observational arm (B), as sketched in Fig. 1A. Despite the fact that randomisation was planned on a 1:1 basis, due to the early termination of patient enrollment, it was not possible to achieve a balanced randomisation as detailed in the below Patients section. For the former, the administration of six doses of DC-based vaccine was planned. The first dose of a freshly prepared vaccine was administered at the end of the cell culture, while the remaining five cryopreserved aliquots were used to prepare the further doses administered every 4 weeks to complete 6 months of therapy. Additionally, about 3 MU IL-2/day were administered subcutaneously for 5 days starting the day after each vaccine dose. Differently, pts afferent to the observational arm underwent clinical and laboratory evaluations according to established time points by the study protocol.

## Results

### Patients

Between 2015 and 2019, a total of 18 eligible melanoma pts were randomly included in this study, 10 of whom in the experimental arm with autologous DC vaccine (arm A), and 8 pts in the observational arm (arm B). Baseline pt characteristics are summarised in Table 1 and Additional file 3 details the trial consort diagram. Median age was 60 years (range 32–78) and 55.6% of pts were females, 6 and 4 pts in arm A and B respectively. Only 3 pts (16.7%) previously underwent adjuvant therapy based on high-dose IFNalfa2b and 55.6% of all enrolled pts developed the first metastasis within 2 years of primitive tumor diagnosis. All pts had an ECOG performance status of 0 and were resected stage IV at study entry (11 pts of stage IV M1a, 5 of stage IV M1b and 2 of stage IV M1c). The BRAF mutation was harbored by 66.7% of pts, 7 and 5 pts in arm A and B respectively. At data cut-off median follow up (FUP) was 24 months (2–64). Overall, median RFS was 5.3 months, 95% confidence interval (CI) 3.2–22.5, but pts receiving DC vaccination have shown a better RFS compared to the observational cohort (median 6.6 months, 95% CI 2.3–not reached (nr) (arm A) vs 5.2 months, 95% CI 2.5–nr (arm B), p = 0.928) (HR = 0.95, 95% CI 0.32–2.84). Intriguingly, favorable trends in arm A, even if not significant, were observed for female pts (median 15.5 months, 95% CI 2.6–nr (female) vs 3.3, 95% CI 2.3–nr (male), pts with less than 60 years (median 22.5 months, 95% CI 2.6–nr (age < 60) vs 4.7 months, 95% CI 2.3–nr (age ≥ 60), and with wild-type BRAF status (median 22.5 months, 95% CI 8.6–nr (BRAF wt) vs 3.8 months, 95% CI 2.3–nr (BRAF mut) (Fig. 1B and Additional file 4). An univariate analysis of RFS on the whole pt cohort is summarized in Additional file 4.

**Table 1:** Patient’s baseline characteristics

Variable	Arm A: Vaccine n = 10 (55.6%) N (%)	Arm B: Observation n = 8 (44.4%) N (%)	Overall n = 18 (100%) N (%)
Age (years)			
< 60	5 (62.5)	3 (37.5)	8 (44.4)
≥ 60	5 (50.0)	5 (50.0)	10 (55.6)
Median (range, IQR)	59 (32–78, 22)	60 (43–76, 18)	60 (32–78, 20)
Mean value (SD)	56.5 (14.4)	59.6 (11.6)	57.9 (13.0)
Gender			
Male	4 (40.0)	4 (50.0)	8 (44.4)
Female	6 (60.0)	4 (50.0)	10 (55.6)
Time from primitive tumour to first metastasis			
> 2 years	5 (50.0)	3 (37.5)	8 (44.4)
≤ 2 years	5 (50.0)	5 (62.5)	10 (55.6)
Stage at study entry (prior to surgery)			
IV M1a	6 (60.0)	5 (62.5)	11 (61.1)
IV M1b	3 (30.0)	2 (25.0)	5 (27.8)
IV M1c	1 (10.0)	1 (12.5)	2 (11.1)
BRAF status			
Wild type	3 (30.0)	3 (837.5)	6 (833.3)
Mutated	7 (70.0)	5 (62.5)	12 (66.7)
Comorbidities			
No	8 (80.0)	7 (87.5)	15 (83.3)
Yes	2 (20.0)	1 (12.5)	3 (16.7)
Previous adjuvant therapy			
No	8 (80.0)	7 (87.5)	15 (83.3)
Yes	2 (20.0)	1 (12.5)	3 (16.7)
Type of adjuvant therapy			
Radiotherapy	0	0	0
Systemic chemotherapy	2 (100)	1 (100)	3 (100)
Immunotherapy	0	0	0
Biological therapy	0	0	0
Other	0	0	0

The sites of relapse also differed between arms, with 4 out of 6 relapsed (R) pts in the control arm developing brain metastases and 1 soft tissue and bone metastases, while soft tissue or locoregional lymph nodes were the most common sites of relapse in pts in the treatment arm (only 1 out of 7 pts had brain metastases and 1 visceral metastasis). The toxicity profile was very favorable as we had no grade 4 events and the few grade 3 events were local reactions at the site of DC vaccine injection and gastrointestinal toxicity due to low dose IL-2 following vaccine injection. Grades 1–2 treatment related adverse events (AEs) were all limited to injection site reactions or fever due to IL-2 therapy. A detailed description of reported AEs is shown in Additional file 5.

### DC product phenotype and potency features

All DC batches were manufactured according to current good manufacturing practice (GMP) guidelines and met the required safety quality control acceptance criteria [16]. The average recovery by number of seeded PBMCs was 3.40% ± 1.1% (mean ± σ) for the DC batches considered in this study. On the last day of culture, in order to prepare vaccine syringes, the DC viability (93.3% ± 1.9%) and purity (64.1% ± 3.0%) were evaluated, additionally the expression of DC-specific maturation markers was performed: CD80 (95.0% ± 5.8%), CD83 (78.0% ± 15.4%), CD86 (98.3% ± 2.7%) and HLA-DR (86.9% ± 8.9%). The potency of each batch produced was evaluated in terms of the ability of DCs to stimulate T lymphocyte proliferation [17]. The analysis performed on six batches out of ten, due to the material shortage, showed an average potency of 56.6% ± 18.0%.

### Immune response assessment and immune-related biomarkers

**Fig. 2:**
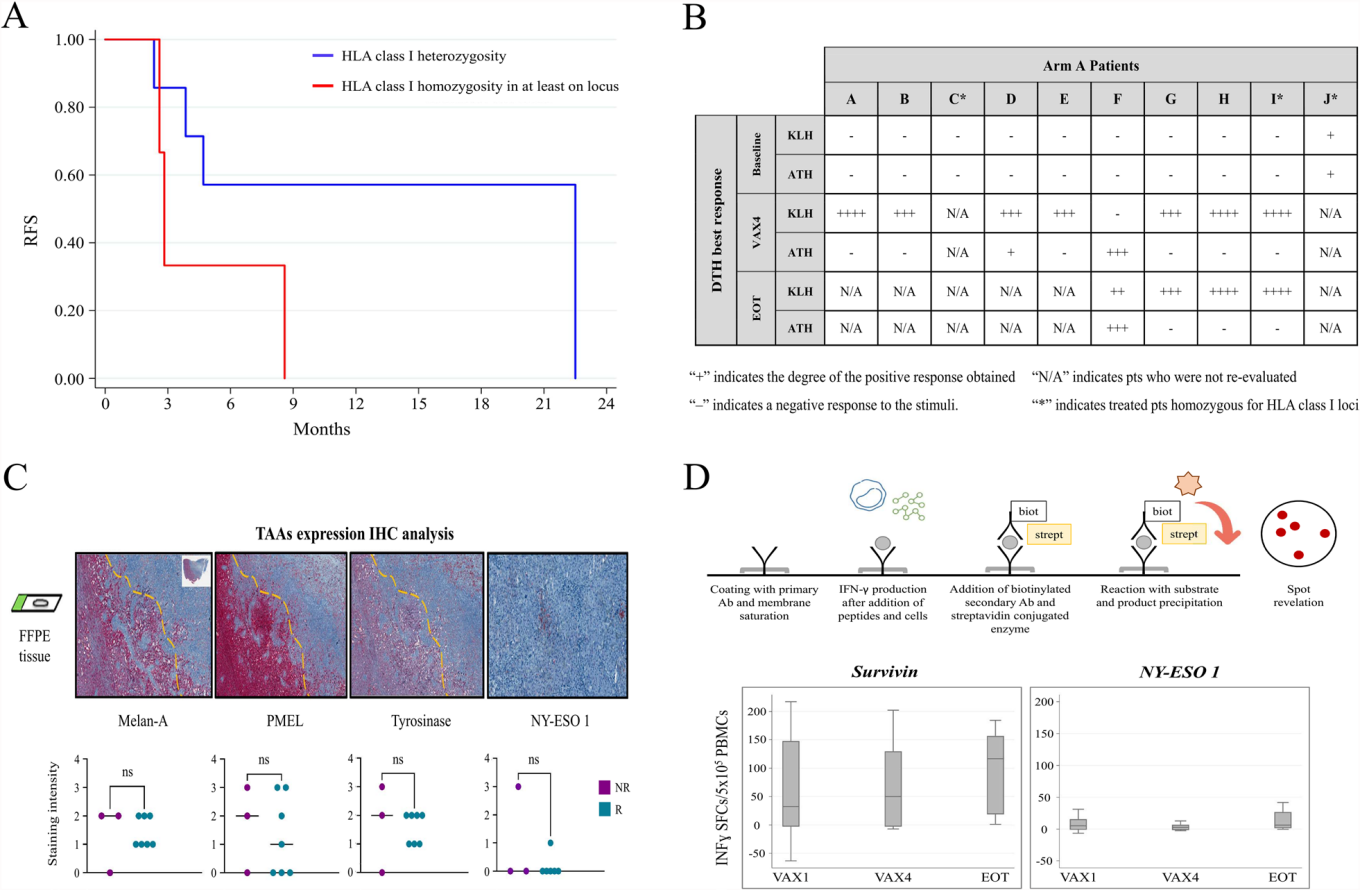
**A** Kaplan–Meier curve of the univariate analysis on RFS of HLA class I allelic heterozygosity/homozygosity distribution in arm A pts. **B** The table shows the DTH in vivo test best response to KLH and ATH in vaccinated pts (in columns from A to J). **C** Representative images of the TAA expression analysis by immunohistochemistry (IHC) on pre-vaccine biopsies collected from arm A pts (upper line). Dot plots with bars represent the staining intensity of each analyzed melanoma specific marker (Melan-A, Pmel, Tyrosinase and Ny-eso1 from left to right). Pts were divided in R and NR. **D** INFγ ELISPOT test steps and graphical representation of the median number of INFγ SFCs on 5 × 10^5^ PBMCs measured at baseline, VAX4 and at the EOT in arm A pts after Survivin and Ny-eso1 peptides stimulation, respectively

To investigate the immunological susceptibility and specific immunological responsiveness of our pt cohort we intersected several assessments. In particular, the determination of heterozygosity at the HLA class I loci (HLA-A, HLA-B or HLA-C) can be considered a marker of susceptibility to immune response and was defined by HLA type analysis. In the entire case study, only 4 pts (3 in arm A and 1 in arm B) had a homozygous haplotype in at least one locus, and all of them had rapid disease progression. Figure 2A shows a univariate analysis of RFS in relation to heterozygosity in HLA class I loci in arm A pts. We found that HLA heterozygous pts had a better RFS than homozygotes (median 22.5 months, 95% CI 2.3–nr vs 2.8 months, 95% CI 2.6–nr, ns).

The Delayed-Type Hypersensitivity (DTH) reaction was used to assess the in vivo immunological response before, during (VAX4) and at the end of treatment (EOT); the data are presented in Fig. 2B. Most of the pts did not show an immunological response at the baseline, while a variable response to keyhole limpet hemocyanin (KLH) and a weak response to autologous tumor homogenate (ATH) were observed in the pts evaluated after the fourth vaccination. For clinical reasons, only four pts were evaluated at the EOT and only one showed a relevant response to ATH. Two of the three pts homozygous for HLA class I loci discontinued vaccine treatment due to disease progression before re-evaluation, while the third did not respond to ATH at EOT. Moreover, tumor biopsies collected before treatment were evaluated for the expression of a panel of TAAs known to be highly expressed in > 80% of melanoma and used to assess the immunological efficacy of the treatment by IFN-ɣ ELISPOT assay. A semiquantitative evaluation was performed by 2 different operators and reviewed by a senior expert pathologist (FL), considering the median percentage of positive cells in five representative fields. Overall, considering the staining intensity (scored as: 1 for weak, 2 for medium and 3 for high intensity, respectively) a lower expression, albeit not significant, of melanoma specific markers was observed on tumor tissue from R pts compared to non relapsed (NR) pts (Melan-A 1.43 ± 0.20 vs. 1.33 ± 0.66, Pmel 1.29 ± 0.52 vs. 1.67 ± 0.88, Tyrosinase 1.57 ± 0.20 vs. 1.67 ± 0.88, Ny-Eso 1 0.17 ± 0.17 vs. 1.00 ± 1.00, mean staining intensity ± SEM) (Fig. 2C). Then, the in vitro antigen-specific immune response analysis performed by means of ELISPOT in arm A pts showed non-significant but relevant levels of increasing reactivity against NY-ESO-1 and survivin after DC vaccination (Fig. 2D).

### Systemic immune modulation related to DC vaccine

**Fig. 3:**
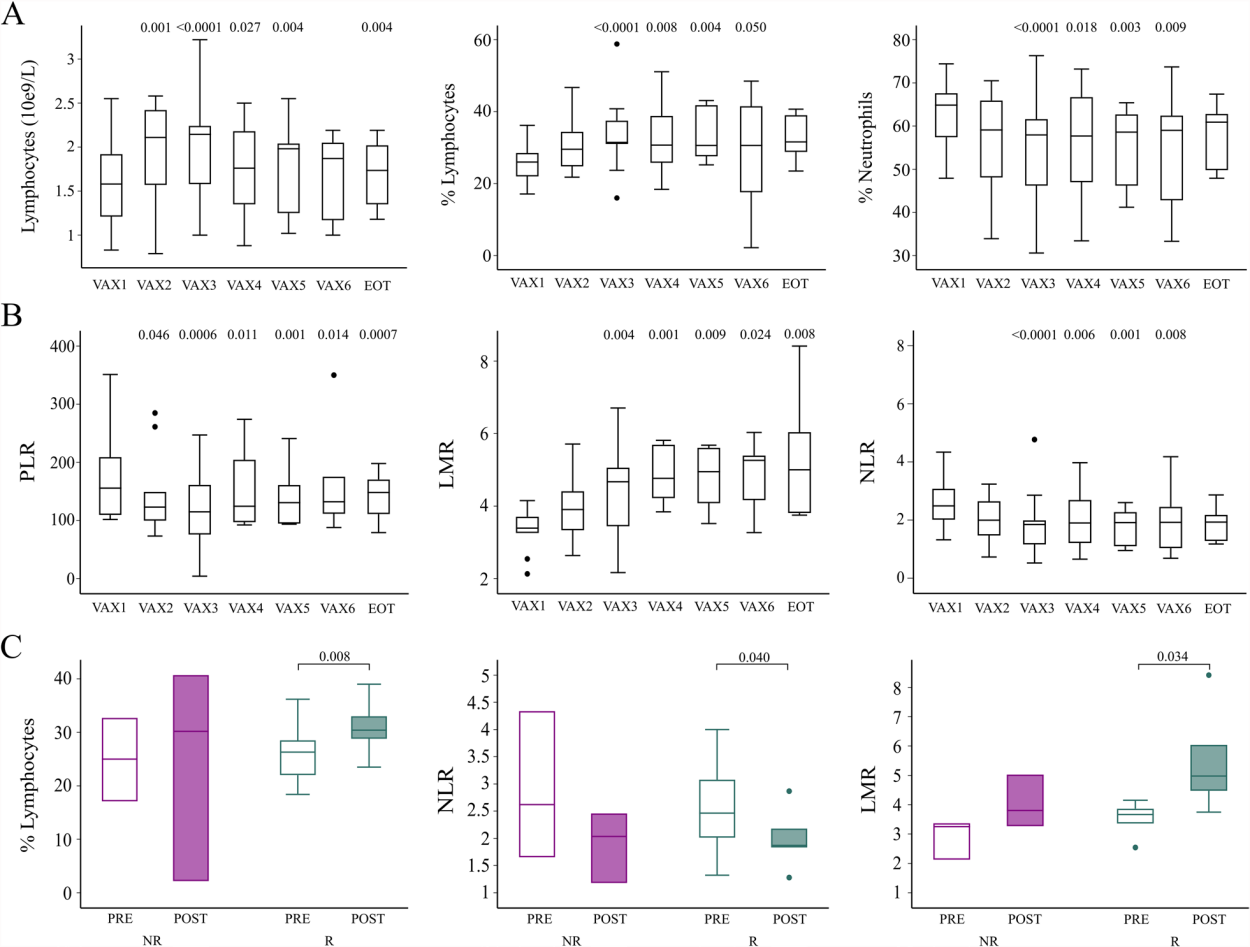
**A** Boxplot of significantly modulated peripheral blood cell biomarkers, collected during treatment in arm A pts, and analyzed by non-parametric ANOVA test for repeated measures. **B** Boxplot showing the tendency of the ratios PLR, LMR and NLR during treatment. **C** Boxes with floating bars represent the lymphocytes percentage, the NLR and the LMR. Statistical analysis was performed with the nonparametric Wilcoxon signed rank test; the exact p value of the comparisons is shown on graphs

The evaluation of peripheral blood component values throughout the treatment period was performed in arm A pts. A significant variation between baseline and different time points was observed in the percentage (increase, p = 0.004) and in the number (10^9^/L) (increase, p = 0.003) of lymphocytes (Fig. 3A), in the percentage of neutrophils (decrease, p = 0.004) and eosinophils (increase, p = 0.003) and in the number (10^9^/L) of eosinophils (increase, p = 0.0005) and basophils (increase, 0.03 ± 0.01, p = 0.049). In addition, also (LMR), neutrophil–lymphocyte ratio (NLR) and platelet-lymphocyte ratio (PLR) were found to significantly change during the treatment (increase, p = 0.019; decrease, p = 0.002; decrease, p = 0.008; respectively) (Fig. 3B). Although not significant, we observed a reduction of the number of platelets and monocytes from the beginning to the EOT (data not shown). The data collected were also studied according to the pt’s outcome. In the cohort of pts that experienced disease relapse, a significant decrease of platelet blood concentration (229.57 ± 54.13 10^9^/L vs 210.20 ± 40.20 10^9^/L, p = 0.034) and NLR (2.53 ± 0.84 vs 2.01 ± 0.58, p = 0.040) was observed between baseline and EOT, respectively. At the same time we found a significant increase in the percentage of lymphocyte (26.36 ± 5.56 vs 30.94 ± 5.69, p = 0.008) and in LMR (3.53 ± 0.51 vs 5.53 ± 1.82, p = 0.034) between baseline and EOT, respectively (Fig. 3C). In NR pts non significant modulation was observed.

### Phenotypic analysis of modulated immune subsets and cytokine profiling

Multi-parametric flow cytometric analysis allowed us to further investigate the overall immunological effects of the treatment. Briefly, a first gate was set, excluding doublets and debris, on physical parameters (FSC and SSC), after which viable cells were selected and additional gates were set to identify immune cell subsets. Monocytes were identified as classical (CD14 + + /CD16-), intermediate (CD14 + + /CD16 +) and non-classical (CD14 + /CD16 + +). In addition, PDL1 expression was evaluated on monocyte subsets. The myeloid derived suppressor cells (MDSCs) subsets were identified as M-MDSC (CD14^+^HLA-DR^−/lo^), eMDSC (Lin^−^HLADR^−^CD33^+^) and PMN-MDSC (CD14^−^CD66b^+^CD11b^+^). Cytotoxic T lymphocytes (CD3 + /CD8 +) were defined as naive (TN, CCR7 + /CD45RA +), central memory (TCM, CCR7 + /CD45RA −), effector memory (TEM, CCR7 −/CD45RA −) and effector (TE, CCR7-/CD45RA +). The subsets of T helper lymphocytes (CD3 + /CD4 +) were defined in the same way.

**Fig. 4:**
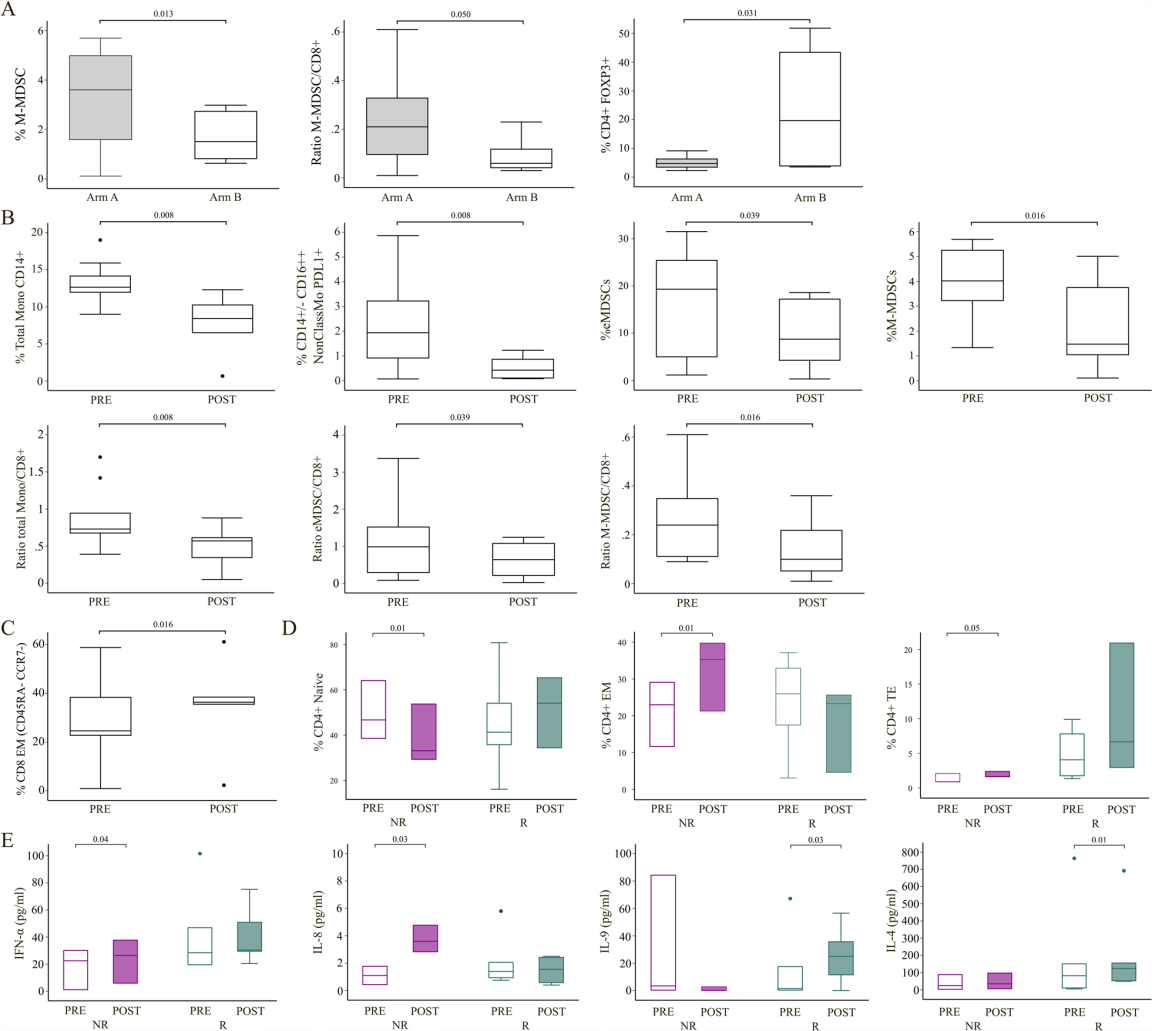
**A** Boxes with floating bars representing the frequency of M-MDSCs, ratio of M-MDSCs on CD8 + lymphocytes and of regulatory T cells (CD4 + FOXP3 +) significantly modulated in arm A pt’s bloodstream compared to arm B pts. **B** Box plots with bars representing the abundance of total circulating monocytes (CD14 +), non classical monocytes (CD14-CD16 +) PDL1 + , eMDSCs and M-MDSC in pre and post-treatment blood samples of arm A pts with below panels representing the ratio between each population and the frequency of CD8 + cells. **C** Box plots representing the frequency of CD8 + EM in arm A pts before and after treatment. **D** Boxes with floating bars relative to CD4 + naive, CD4 + EM and CD4 + TE subsets frequencies in R and NR arm A pts before and after treatment. **E** Box plots with bars represent the concentration levels (pg/mL) of INFα, IL-8, IL-9 and IL-4 in vaccinated pts. Statistical analysis was performed with the nonparametric Wilcoxon signed rank test; the exact p value of the comparisons is shown on graphs

Comparing values at baseline between arm A (vaccine) and B (observation), the frequency of M-MDSCs (3.95 ± 1.53 (A) vs 1.73 ± 1.09 (B), p = 0.013) and the ratio of M-MDSC to CD8 (0.27 ± 0.16 (A) vs 0.10 ± 0.08 (B), p = 0.05) are significantly higher in arm A than in arm B. However, pts in arm B had a higher frequency of CD4 + /FOXP3 + cells than pts on treatment (4.86 ± 2.20 (A) vs 23.66 ± 23.94 (B), p = 0.031) (Fig. 4A).

Immunophenotypic analysis was performed throughout the treatment period in arm A pts. A significant decrease was observed at EOT compared to the baseline in the frequency (%) of eMDSCs (19.30 (pre) vs 8.75 (post), p = 0.039), M-MDSCs (4.02 (pre) vs 1.47 (post), p = 0.016), total CD14 + (12.65 (pre) vs 8.43 (post), p = 0.008), classical monocytes (11.20 (pre) vs 7.80 (post), p = 0.008), intermediate monocytes (0.76 (pre) vs 0.43 (post) p = 0.039) and non-classical monocytes expressing PDL1 (1.94 (pre) vs 0.42 (post), p = 0.008). The ratios of total (0.73 (pre) vs 0.57 (post), p = 0.008), classical (0.64 (pre) vs 0.53 (post), p = 0.008) and intermediate (0.05 (pre) vs 0.03 (post), p = 0.039) monocytes to CD8 showed a decrease, as well as the ratio of eMDSC (0.98 (pre) vs 0.64 (post), p = 0.039) and M-MDSC to CD8 (0.24 (pre) vs 0.10 (post), p = 0.016) (Fig. 4B). Moreover, from the lymphoid subsets analysis an increase was observed in the frequency of CD8 EM (24.60 (pre) vs 36.35 (post), p = 0.016) after treatment (Fig. 4C). When we analyzed data from arm A pts considering the clinical outcome we found a significant decrease in the frequency of PMN-MDSCs in NR pts between pre and post-treatment (96.40 (pre) vs 94.30 (post), p = 0.033, data not shown. We also considered the variation over time of serum pro-inflammatory cytokine and we observed a general, but not significant, increase in GM-CSF, IFN-α, IFN-γ, IL-4, IL-5, IL-8, IL-9, IL-10, IL-12p70, IL17A and TNF-α levels between baseline and the fourth vaccination or EOT. Otherwise, pts in arm B showed a significant decrease in IFN-α (24.50 ± 13.11, p = 0.045) and IL-6 (3.95 ± 2.91, p = 0.045) levels between FUP1 and FUP2 timepoints (data not shown).

Moreover, we found a significant decrease of CD4 + Naïve T cells and a concomitant increase in CD4 + Effector Memory (CD4 + EM), CD4 + Terminal Effector (CD4 + TE), and in IFN-α (17.87 ± 15.31 (pre) vs 23.33 ± 16.42 (post), p = 0.038), and IL-8 (1.10 ± 0.70 (pre) vs 3.73 ± 1.01 (post), p = 0.030) serum levels in post treatment (EOT) samples from NR vaccinated pts. In R pts, a significant post treatment increase was observed in the serum concentration of IL-4 (182.47 ± 291.35 (pre) vs 214.64 ± 271.00 (post), p = 0.009) and IL-9 (14.67 ± 26.65 NR (pre) vs 25.76 ± 21.98 (post), p = 0.032) (Fig. 4D, E). No other significant modulations were highlighted in R or in arm B pts.

### Baseline intratumoral immune cells subsets frequency and PDL1 expression by tumor cells marks R from NR patients

**Fig. 5:**
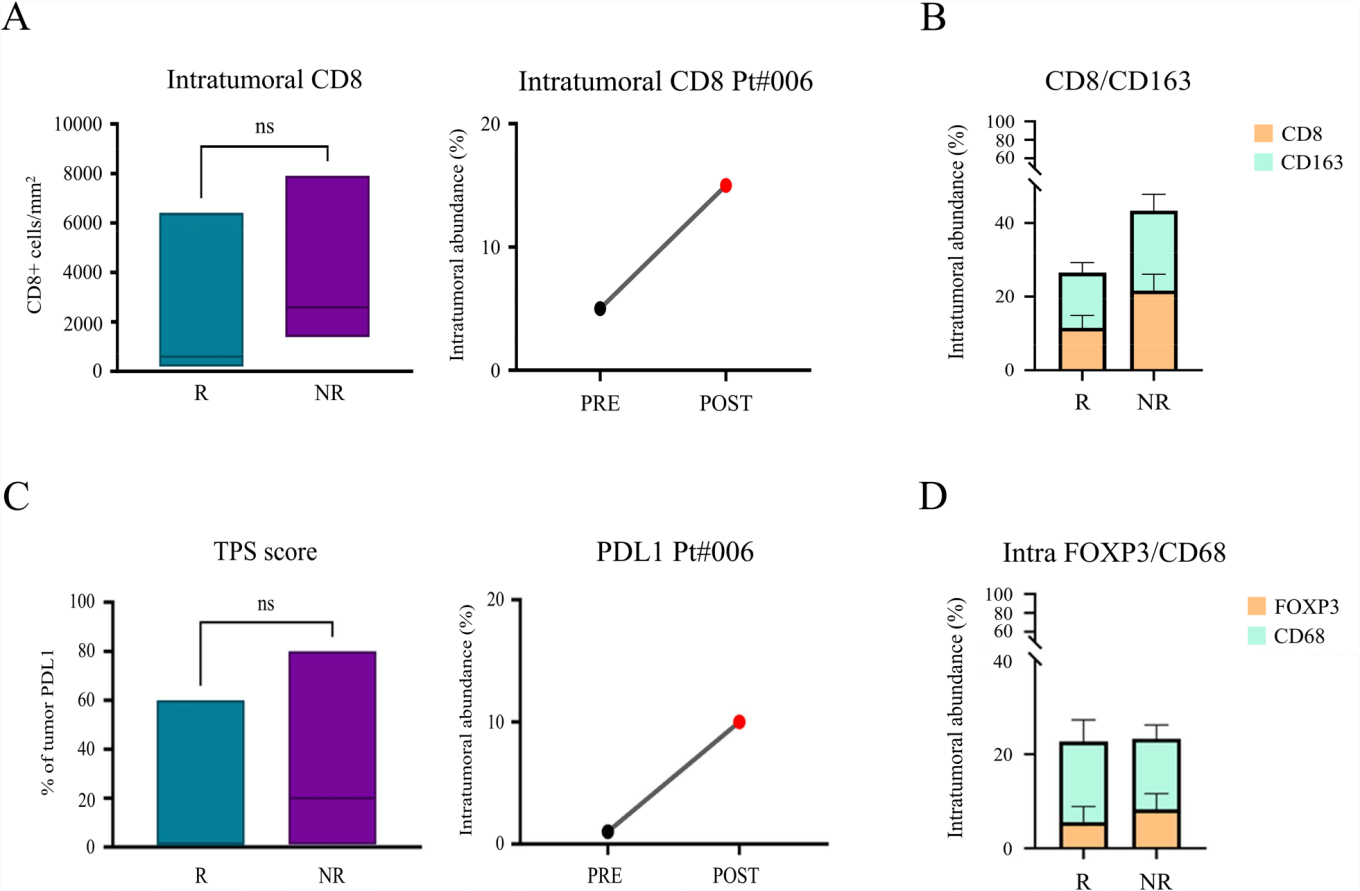
**A** The number of intratumoral CD8 + T cells per mm^2^ in pre-treatment biopsies of arm A pts (p = 0.2667) is plotted in graphs. Right graph shows the increment of CD8 expression after treatment in Pt#006 (R pt) biopsies. **B** The abundance of intratumoral CD8 + and CD163 + cells in pre- treatment biopsies is plotted in graphs. **C** Differences in PDL1 + tumor cells are illustrated in the graph as the percentage of PDL1 expressing tumor cells on the total tumor cell number (TPS 17.71 ± 9.007 vs. 33.67 ± 23.81, R vs NR, p = 0.5417). Right graph shows the increment of PDL1 expression after treatment in Pt#006 (R pt) biopsies. **D** The abundance of intratumoral FOXP3 + and CD68 + cells in pre-treatment biopsies is plotted in graph

At the tumor site, analyzing tumor biopsies collected before treatment, we found an increased number, albeit not significant, of intratumoral CD8 + T cells in baseline tissue biopsies in NR (3958 ± 2003 positive cells/mm^2^) compared to R (1925 ± 867.7 positive cells/mm^2^) pts (Fig. 5A). Intriguingly, for pt #006 (R) for whom a post-therapy biopsy was available we observed an increment of intratumoral CD8 + cells after treatment (Fig. 5A, right panel).

To gain further insight into the intratumoral immune cell landscape of DC vaccinated pts before therapy, we performed a double staining on the same tissue section of CD8 + and CD163 + cells, allowing the relative abundance of these immune cells in the tissue to be distinguished. The frequency (%) of CD163 + (15.00 ± 2.67 vs. 21.67 ± 4.41, p = 0.2917) and CD8 + (11.57 ± 3.32 vs. 21.67 ± 4.41, p = 0.1583) in pre-vaccine biopsies differed, although not significantly, among R and NR pts (Fig. 5B). PD-L1 expression in tumor tissue was quantified as the percentage of live tumor cells that exhibited specific cell surface staining of any intensity in a section containing at least 100 evaluable tumor cells, with ≥ 5% defined as positive staining, as previously described [18]. In particular, tumor proportion score (TPS) was calculated as PDL1 + tumor cells/ total n. tumor cell*100. We found a higher percentage of PDL1 + tumor cells in NR pts compared to R pts in pre-vaccination tumor biopsies (TPS 17.71 ± 9.007 vs. 33.67 ± 23.81, R vs NR, p = 0.5417). Of note, in pt #006 (R) for whom a post-therapy biopsy was available we observed an increase in PDL1 expression after treatment (Fig. 5C). In addition, a second double staining involving FOXP3 and CD68 was performed on a second tissue section. The frequency (%) of intratumoral FOXP3 + (5.57 ± 3.32 vs. 8.33 ± 3.33, R vs NR, p = 0.1667) and CD68 + (17.14 ± 4.61 vs. 15.00 ± 2.89, R vs NR, p = 0.9061) cells did not differ significantly between the groups of pts (Fig. 5D).

## Discussion

Schadendorf et al. conducted the first large randomised DC-based vaccine trial in metastatic melanoma, which did not show a survival benefit compared to dacarbazine. However, a recent Bayesian network meta-analysis by Lau P. et al. comparing all vaccine types in melanoma, concluded that OS data support autologous DC-based vaccines as an option in the metastatic setting. In addition, a meta-regression model showed a strong interaction between OS benefit and gender, with the proportion of female vaccinated pts positively correlated with survival benefit. The mortality risk for vaccination decreased by 2.74% per 1.00% increase in the number of female pts (95% CI 5.23 to 0.38) [19, 20]. In the adjuvant setting, following preliminary positive results of Bol KF in resected stage III melanoma in 2016, the phase III study published in 2024 did not meet the primary endpoint. However, the study was halted as the placebo arm had become unethical [12, 13].

Our smaller randomised phase II study in higher-risk melanoma pts, was stopped for similar reasons. Despite the small sample size, we observed an RFS benefit in the treatment arm compared to the control arm, with more consistent results in pts younger than 60 years and in females. In agreement with findings in gender-based oncology, younger women showed better RFS than older men, highlighting the importance of considering gender and age in therapeutic outcomes and trial design [20, 21].

In addition to clinical evidence, characterising the immune profile is crucial to better define the “cancer-immune set point” and to fully benefit from cancer immunotherapy, which aims to activate an anti-cancer response that can target multiple mechanisms, rather than focusing on a single target as in conventional therapies [22]. We conducted a detailed analysis of the immune profile of melanoma pts receiving a DC vaccine pulsed with autologous tumor lysate as adjuvant therapy that met quality control specifications, confirming that all pts were treated to a defined standard of safety and cell potency in compliance with cGMP guidelines [16]. The aim was to elucidate the mechanism of action of our therapy and to precisely identify which pts are most likely to benefit from it, supporting DC-based vaccines as a low toxicity option for adjuvant therapy.

In our study, we profiled the HLA class I and class II haplotype correlating this with the pt’s clinical outcome considering the potential as a marker of immune response susceptibility. We observed that NR treated pts had maximal heterozygosity at HLA class I loci (HLA-A, HLA-B or HLA-C), an HLA-A02 profile and, in two-thirds of cases, an HLA-B35 supertype, which has previously been associated with favourable clinical outcomes [23–25]. On the other hand, our data confirm that anti-tumor efficacy is associated, although not significantly, with the expression of TAAs in collected pre-treatment biopsies, particularly in NR pts. We observed that a robust TAAs expression together with a favorable immune infiltrate contexture inside the tumor (high intratumoral CD8 + cells/low FOXP3 + Treg and CD163 + myeloid cells) improve the vaccine ability to protect pts from cancer recurrence. Furthermore, in one pt we observed, similarly to what was seen in a previously examined advanced melanoma cohort [26], that the vaccine increases the expression of tumoral PDL1 making the tumor more sensitive to ICIs. The immune profile of the tumor is also influenced by the BRAF mutation and its pathway, a key target in melanoma immunotherapy [27]. In our study population, wild-type BRAF was correlated with a protective effect of the vaccine and a significantly improved RFS. To further determine the specific anti-tumor immune response, pts were evaluated using the DTH test. Although an in vivo specific response to the ATH used to pulse the DCs during the manufacturing process could not be demonstrated, the results suggest that the therapy triggered an activation of the immune system that was previously absent at baseline. To gain a clearer understanding of the nature of the induced immune response, an analysis of the immune profile was conducted [28, 29]. The changes in serum cytokines and peripheral blood components further underscore that our cellular therapy effectively enhanced the activity of the immune system. The increase in lymphocytes, eosinophils and basophils, coupled with the decrease in neutrophils and PLR observed in pts during treatment and compared to those in the observation arm, is commonly associated with an immune-inflamed phenotype and a potentially improved prognosis in advanced cancer and in particular in melanoma. This is further supported by the increase, albeit not significant, in cytokines associated with T cell-mediated immune responses [30–33]. In line with this, the immunophenotypic profiling of circulating immune cells revealed that treated pts experienced a decrease in myeloid cell subtypes over time (i.e. e-MDSCs, M-MDSCs and PDL1 + non-classical monocytes); and a simultaneous increase in CD8 + TEM lymphocytes, a key player in the anti-tumor response especially when generated at high frequencies. Then focusing on NR vaccinated pts we noticed a shift also in the T helper CD4 + cells with a significant decrease in the naive compartment in favour of an increase in the CD4 + TEM and TE subpopulation described as essential in initiating and sustaining among others the anti-PD1 induced systemic response [34, 35]. Concomitantly, a significant pro-inflammatory cytokine profile was observed in NR pts, with an increase in IFN-α and IL-8 after treatment. Intriguingly, we also observed a dynamic systemic immune modulation in treated R pts with an increase in lymphocytes, LMR, IL-4 and IL-9 and a decrease in platelets and NLR, suggesting that vaccination had a positive impact on the immune response in some pts, but not enough to achieve a durable clinical benefit [36–38]. Then, considering the untreated pts cohort, a significant reduction in IFN-α, commonly associated with an anti-cancer response [39], and IL-6, a cytokine with a more enigmatic but protective role against cancer [40], was found over time during follow-up. Overall, evidence supports that the proportion of certain immune cell types is associated with the response to immunotherapy in cancer, including melanoma. While phase III trials in stage III melanoma have suggested that adjuvant immunotherapy improves RFS, the OS benefit has yet to be confirmed [2–6, 41, 42] and no specific subpopulations have been identified that alone can predict outcomes. Our study has evident limitations, indeed despite the statistical significance of some data reported herein, the pts cohort was small and the rapid advances of adjuvant ICIs as frontline treatment in melanoma has abruptly halted the enrolment of pts eligible for this experimental autologous cell therapy. In spite of these limitations, this study provides a more comprehensive view of the immunological processes involved in the response to anti-tumor DC vaccination in the melanoma adjuvant setting, opening new perspectives for clinical research challenges and combinatorial immunotherapy regimens aimed at personalized cancer treatment.

## Conclusions

This study highlights the potential of DC-based vaccines as a well-tolerated adjuvant therapy for melanoma, particularly benefiting younger individuals and females in the RFS. Although limited by the growing preference for ICIs and sample size, which makes it difficult to generate robust and statistically significant data, it provides valuable insights into the immunological factors that influence vaccine efficacy. We identified key factors that may improve therapeutic precision and help stratify patients in future trials. The associations between wild-type BRAF, tumour-infiltrating lymphocytes and immune cell composition highlight the importance of a personalized approach to immunotherapy. With reduced toxicity and the ability to induce a diverse systemic anti-tumour response, DC vaccines offer advantages over current adjuvant treatments. Although larger trials, better understanding of gender and age dynamics, and combination strategies are needed, this research paves the way for the advancement of personalized immunotherapy in melanoma.

## Supplementary Information

Additional file 1. Methods

Additional file 2. Details of reagents, kits, softwares and instruments used in the study

Additional file 3. CONSORT Flow diagram of the clinical trial

Additional file 4. The table shows the univariate analysis of RFS of the entire pts cohort for age, gender and Braf gene status.Kaplan-Meier curve of the univariate analysis of RFS by age.Kaplan-Meier curve of the univariate analysis of RFS by gender.Kaplan-Meier curve of the univariate analysis of RFS by Braf gene status

Additional file 5. Number of patients with at least 1 cycle of treatment with reported adverse events

## Data Availability

The datasets generated and/or analyzed during the current study are available from the corresponding author on reasonable request.

## Abbreviations

AE: Adverse events
ATH: Autologous tumor homogenate
CI: Confidence interval
DC: Dendritic cell
DTH: Delayed-type hypersensitivity
EOT: End of treatment
FUP: Follow up
GMP: Good manufacturing practise
HLA: Human leukocyte antigens
ICI: Immune checkpoint inhibitors
KLH: Keyhole limpet hemocyanin
LMR: Lymphocyte-monocyte ratio
NLR: Neutrophil–lymphocyte ratio
nr: Not reached
NR: Not relapsed
ns: Not significant
OS: Overall survival
PLR: Platelet-lymphocyte ratio
Pt: Patient
R: Relapsed
RFS: Relapse-free survival
TAA: Tumor-associated antigens
TPS: Tumor proportion score
VAX: Vaccine

## References

[1] Mocellin S, Pasquali S, Rossi CR, Nitti D. Interferon alpha adjuvant therapy in patients with high-risk melanoma: a systematic review and meta-analysis. J Natl Cancer Inst. 2010;102(7):493–501. 20179267 10.1093/jnci/djq009

[2] Eggermont AMM, Chiarion-Sileni V, Grob J-J, et al. Adjuvant ipilimumab versus placebo after complete resection of high-risk stage III melanoma (EORTC 18071): a randomised, double-blind, phase 3 trial. Lancet Oncol. 2015;16(5):522–30. 25840693 10.1016/S1470-2045(15)70122-1

[3] Eggermont AMM, Chiarion-Sileni V, Grob J-J, et al. Prolonged survival in stage III melanoma with ipilimumab adjuvant therapy. N Engl J Med. 2016;375(19):1845–55. 27717298 10.1056/NEJMoa1611299 PMC5648545

[4] Larkin J, Del Vecchio M, Mandalá M, et al. Adjuvant nivolumab versus ipilimumab in resected stage III/IV melanoma: 5-year efficacy and biomarker results from CheckMate 238. Clin Cancer Res. 2023;29(17):3352–61. 37058595 10.1158/1078-0432.CCR-22-3145 PMC10472092

[5] Long GV, Hauschild A, Santinami M, et al. Final results for adjuvant dabrafenib plus trametinib in stage III melanoma. N Engl J Med. 2024;391(18):1709–20. 38899716 10.1056/NEJMoa2404139

[6] Eggermont AM, Kicinski M, Blank CU, et al. Seven-year analysis of adjuvant pembrolizumab versus placebo in stage III melanoma in the EORTC1325 / KEYNOTE-054 trial. Eur J Cancer. 2024;211: 114327. 39288737 10.1016/j.ejca.2024.114327

[7] Livingstone E, Zimmer L, Hassel JC, Fluck M, Eigentler TK, Loquai C, et al. Adjuvant nivolumab plus ipilimumab or nivolumab alone versus placebo in patients with resected stage IV melanoma with no evidence of disease (IMMUNED): final results of a randomised, double-blind, phase 2 trial. Lancet. 2022;400(10358):1117–29. 36099927 10.1016/S0140-6736(22)01654-3

[8] Najafi S, Mortezaee K. Advances in dendritic cell vaccination therapy of cancer. Biomed Pharmacother. 2023;164: 114954. 37257227 10.1016/j.biopha.2023.114954

[9] Rodriguez J, Castañón E, Perez-Gracia JL, et al. A randomized phase II clinical trial of dendritic cell vaccination following complete resection of colon cancer liver metastasis. J Immunother Cancer. 2018;6(1):96. 30268156 10.1186/s40425-018-0405-z PMC6164167

[10] Nava S, Lisini D, Frigerio S, Bersano A. Dendritic cells and cancer immunotherapy: the adjuvant effect. Int J Mol Sci. 2021;22(22):12339. 34830221 10.3390/ijms222212339 PMC8620771

[11] Boudewijns S, Bol KF, Schreibelt G, et al. Adjuvant dendritic cell vaccination induces tumor-specific immune responses in the majority of stage III melanoma patients. Oncoimmunology. 2016;5(7): e1191732. 27622047 10.1080/2162402X.2016.1191732 PMC5006921

[12] Bol KF, Aarntzen EHJG, Hout FEM, et al. Favorable overall survival in stage III melanoma patients after adjuvant dendritic cell vaccination. Oncoimmunology. 2016;5(1):e1057673. 26942068 10.1080/2162402X.2015.1057673 PMC4760342

[13] Bol KF, Schreibelt G, Bloemendal M, et al. Adjuvant dendritic cell therapy in stage IIIB/C melanoma: the MIND-DC randomized phase III trial. Nat Commun. 2024;15(1):1632. 38395969 10.1038/s41467-024-45358-0 PMC10891118

[14] de Rosa F, Ridolfi L, Fiammenghi L, et al. Dendritic cell vaccination for metastatic melanoma: a 14-year monoinstitutional experience. Melanoma Res. 2017;27(4):351–7. 28654547 10.1097/CMR.0000000000000356

[15] Ridolfi L, de Rosa F, Fiammenghi L, et al. Complementary vaccination protocol with dendritic cells pulsed with autologous tumor lysate in patients with resected stage III or IV melanoma: protocol for a phase II randomised trial (ACDC Adjuvant Trial). BMJ Open. 2018;8(8): e021701. 30082356 10.1136/bmjopen-2018-021701 PMC6078243

[16] Granato AM, Pancisi E, Piccinini C, et al. Dendritic cell vaccines as cancer treatment: focus on 13 years of manufacturing and quality control experience in advanced therapy medicinal products. Cytotherapy. 2024;26:1547. 39046388 10.1016/j.jcyt.2024.07.005

[17] Carloni S, Piccinini C, Pancisi E, et al. Potency assessment of dendritic cell anticancer vaccine: validation of the co-flow DC assay. Int J Mol Sci. 2021;22(11):5824. 34072360 10.3390/ijms22115824 PMC8198694

[18] Placke J-M, Soun C, Bottek J, et al. Digital quantification of tumor PD-L1 predicts outcome of PD-1-based immune checkpoint therapy in metastatic melanoma. Front Oncol. 2021;11: 741993. 34621681 10.3389/fonc.2021.741993 PMC8491983

[19] Schadendorf D, Ugurel S, Schuler-Thurner B, et al. Dacarbazine (DTIC) versus vaccination with autologous peptide-pulsed dendritic cells (DC) in first-line treatment of patients with metastatic melanoma: a randomized phase III trial of the DC study group of the DeCOG. Ann Oncol. 2006;17(4):563–70. 16418308 10.1093/annonc/mdj138

[20] Lau P, Shen M, Ma F, et al. A Bayesian network meta-analysis of comparison of cancer therapeutic vaccines for melanoma. J Eur Acad Dermatol Venereol. 2021;35(10):1976–86. 34077578 10.1111/jdv.17437 PMC8518424

[21] Hall M, Krishnanandan VA, Cheung MC, et al. An evaluation of sex- and gender-based analyses in oncology clinical trials. J Natl Cancer Inst. 2022;114(8):1186–91. 35477781 10.1093/jnci/djac092 PMC9360459

[22] Chen DS, Mellman I. Elements of cancer immunity and the cancer-immune set point. Nature. 2017;541(7637):321–30. 28102259 10.1038/nature21349

[23] Chowell D, Morris LGT, Grigg CM, et al. Patient HLA class I genotype influences cancer response to checkpoint blockade immunotherapy. Science. 2018;359(6375):582–7. 29217585 10.1126/science.aao4572 PMC6057471

[24] Ivanova M, Shivarov V. HLA genotyping meets response to immune checkpoint inhibitors prediction: a story just started. Int J Immunogenet. 2021;48(2):193–200. 33112034 10.1111/iji.12517

[25] Sabbatino F, Liguori L, Polcaro G, et al. Role of human leukocyte antigen system as a predictive biomarker for checkpoint-based immunotherapy in cancer patients. Int J Mol Sci. 2020;21(19):7295. 33023239 10.3390/ijms21197295 PMC7582904

[26] Bulgarelli J, Tazzari M, Granato AM, et al. Dendritic cell vaccination in metastatic melanoma turns “Non-T Cell Inflamed” into “T-Cell Inflamed” tumors. Front Immunol. 2019;10:2353. 31649669 10.3389/fimmu.2019.02353 PMC6794451

[27] Teixido C, Castillo P, Martinez-Vila C, Arance A, Alos L. Molecular markers and targets in melanoma. Cells. 2021;10(9):2320. 34571969 10.3390/cells10092320 PMC8469294

[28] Roger I, Montero P, Pérez-Leal M, Milara J, Cortijo J. Evaluation of delayed-type hypersensitivity to antineoplastic drugs-an overview. Cancers (Basel). 2023;15(4):1208. 36831549 10.3390/cancers15041208 PMC9954236

[29] Nagai H, Karube R. Delayed-type hypersensitivity: an excellent indicator of anti-tumor immunity with wilms’ tumor 1 (WT1) dendritic cell vaccine therapy. Cureus. 2023;15(11): e49221. 38143707 10.7759/cureus.49221 PMC10739387

[30] Grisaru-Tal S, Rothenberg ME, Munitz A. Eosinophil-lymphocyte interactions in the tumor microenvironment and cancer immunotherapy. Nat Immunol. 2022;23(9):1309–16. 36002647 10.1038/s41590-022-01291-2 PMC9554620

[31] Poto R, Gambardella AR, Marone G, et al. Basophils from allergy to cancer. Front Immunol. 2022;13:1056838. 36578500 10.3389/fimmu.2022.1056838 PMC9791102

[32] Ward MP, Kane LE, Norris LA, et al. Platelets, immune cells and the coagulation cascade; friend or foe of the circulating tumour cell? Mol Cancer. 2021;20(1):59. 33789677 10.1186/s12943-021-01347-1 PMC8011144

[33] Teijeira A, Garasa S, Ochoa MC, et al. IL8, neutrophils, and NETs in a collusion against cancer immunity and immunotherapy. Clin Cancer Res. 2021;27(9):2383–93. 33376096 10.1158/1078-0432.CCR-20-1319

[34] Borst J, Ahrends T, Bąbała N, Melief CJM, Kastenmüller W. CD4+ T cell help in cancer immunology and immunotherapy. Nat Rev Immunol. 2018;18(10):635–47. 30057419 10.1038/s41577-018-0044-0

[35] Kagamu H, Kitano S, Yamaguchi O, et al. CD4+ T-cell immunity in the peripheral blood correlates with response to anti-PD-1 therapy. Cancer Immunol Res. 2020;8(3):334–44. 31871122 10.1158/2326-6066.CIR-19-0574

[36] Kwaśniak K, Czarnik-Kwaśniak J, Maziarz A, et al. Scientific reports concerning the impact of interleukin 4, interleukin 10 and transforming growth factor β on cancer cells. Cent Eur J Immunol. 2019;44(2):190–200. 31530989 10.5114/ceji.2018.76273 PMC6745546

[37] Vinokurova D, Apetoh L. The emerging role of IL-9 in the anticancer effects of anti-PD-1 therapy. Biomolecules. 2023;13(4):670. 37189417 10.3390/biom13040670 PMC10136177

[38] Bilen MA, Martini DJ, Liu Y, et al. The prognostic and predictive impact of inflammatory biomarkers in patients who have advanced-stage cancer treated with immunotherapy. Cancer. 2019;125(1):127–34. 30329148 10.1002/cncr.31778

[39] Waldmann TA. Cytokines in cancer immunotherapy. Cold Spring Harb Perspect Biol. 2018;10(12):a028472. 29101107 10.1101/cshperspect.a028472 PMC6280701

[40] Orange ST, Leslie J, Ross M, Mann DA, Wackerhage H. The exercise IL-6 enigma in cancer. Trends Endocrinol Metab. 2023;34(11):749–63. 37633799 10.1016/j.tem.2023.08.001

[41] Weber JS, Carlino MS, Khattak A, et al. Individualised neoantigen therapy mRNA-4157 (V940) plus pembrolizumab versus pembrolizumab monotherapy in resected melanoma (KEYNOTE-942): a randomised, phase 2b study. Lancet (London, England). 2024;403(10427):632–44. 38246194 10.1016/S0140-6736(23)02268-7

[42] Amaral T, Ottaviano M, Arance A, et al. ESMO Guidelines Committee. Cutaneous melanoma: ESMO Clinical Practice Guideline for diagnosis, treatment and follow-up. Ann Oncol. 2025;36(1):10–30. 39550033 10.1016/j.annonc.2024.11.006 PMC7618628

[43] MIATA guidelines. http://miataproject.org/. Accessed 28 Aug 2024.

[44] Janetzki S, Britten CM, Kalos M, et al. “MIATA”—minimal information about T cell assays. Immunity. 2009;31(4):527–8. 19833080 10.1016/j.immuni.2009.09.007 PMC3762500

[45] Ridolfi L, Petrini M, Granato AM, et al. Low-dose temozolomide before dendritic-cell vaccination reduces (specifically) CD4+CD25++Foxp3+ regulatory T-cells in advanced melanoma patients. J Transl Med. 2013;11:135. 23725550 10.1186/1479-5876-11-135 PMC3698134

[46] European Pharmacopoeia. https://pheur.edqm.eu/home. Accessed 28 Aug 2024.

[47] Bulgarelli J, Piccinini C, Petracci E, et al. Radiotherapy and high-dose interleukin-2: clinical and immunological results of a proof of principle study in metastatic melanoma and renal cell carcinoma. Front Immunol. 2021;12: 778459. 34777395 10.3389/fimmu.2021.778459 PMC8578837

[48] Darmon-Novello M, Adam J, Lamant L, et al. Harmonization of programmed death-ligand 1 immunohistochemistry and mRNA expression scoring in metastatic melanoma: a multicentre analysis. Histopathology. 2022;80(7):1091–101. 35322452 10.1111/his.14651

