# Supplementary material for: Adjuvant dendritic cell-based immunotherapy in melanoma: insights into immune cell dynamics and clinical evidence from a phase II trial
Source: J Transl Med. 2025 Apr 18;23:455. doi: 10.1186/s12967-025-06403-8 (PMC12007200; doi:10.1186/s12967-025-06403-8)
Supplement: Supplementary file 1 — Additional file 1. Methods [file 12967_2025_6403_MOESM1_ESM.docx]

**Materials and methods**

All reagents, kits, softwares and instruments used in this study are listed in Supplementary Table 1. For biological sample collection and *ex vivo* immunological response evaluation by ELISPOT assay we followed Minimal Information About T cell Assays (MIATA) guidelines.^43,44^

***Biological sample collection***

Whole blood was obtained by venipuncture using ethylenediaminetetraacetic acid (EDTA) tubes for peripheral blood mononuclear cells (PBMCs) isolation via lymphocyte separation media gradient centrifugation, and non-coagulant tubes for serum collection. Samples were processed by IRST biobank personnel within 1 hour (h) of blood sampling. PBMCs were cryopreserved at 5-20x10^6^ PBMCs per vial into the vapor phase of liquid nitrogen, while 1 mL of serum sample per tube were stored at -80°C, until use. PBMC and serum samples were longitudinally collected in pts enrolled in arm A at the first (VAX1) and fourth (VAX4) vaccine treatment cycle and at the EOT. Similarly, from pts in arm B, whenever possible, PBMC and serum samples were collected every 12 weeks after the enrollment (FUP1 and FUP2). For all pts, tumor biopsies were also collected before therapy for IHC analysis, as well as clinical and demographic data (white blood cell count, absolute lymphocyte, neutrophil, monocyte and platelet counts) were collected during the trial.

***Patient’s HLA Genotyping***

DNA was automatically extracted from 1x10^6^ PBMCs with the Maxwell® RSC Whole Blood DNA Kit and the Maxwell® RSC instrument following manufacturer's instructions. DNA quality and quantity were assessed by the NanoDrop™ Spectrophotometer and the HLA typing was performed using PCR sequence-specific oligonucleotide (SSO) by means of the Luminex technology. Briefly, the Lifecodes assay for HLA-SSO typing was used and the manufacturer procedure, based on the hybridization of biotin-labeled single stranded PCR products to SSO probes, was followed. These probes are designed so that each probe hybridizes to a complementary region in the amplified DNA. After hybridization, R-Phycoerythrin Conjugated Streptavidin (SA-PE) is added, which binds to any captured DNA. The Luminex Flow Analyser achieves detection by determining the fluorescent intensity of PE (Phycoerythrin) on each microsphere. Finally, the assignment of the HLA alleles is based on the reaction pattern of the various beads compared to patterns with known HLA alleles. All kits and instruments used are listed in Supplementary Table 1.

***DC generation and vaccine preparation***

DCs were obtained from pts-derived PBMCs in compliance with current GMP guidelines, as already described. ^45^ PBMCs were cultured with CellGro DC Medium at 10×10^6^ cells/mL for 2 h. Then, non-adherent cells were removed from the culture, while adherent cells were cultured in CellGro DC Medium added with recombinant human (rh) interleukin (IL)-4 and rh granulocyte-macrophage colony-stimulating factor (GM-CSF). On the sixth day, 90% of the DC culture was pulsed with ATH (100 mg/mL), while the remaining 10% was pulsed with Immucothel (KLH). On the seventh day, the culture medium was discarded and the cells were incubated in maturation CellGro DC Medium added with rh IL-6, tumor necrosis factor-α (rh TNFα), rh IL-1β, and prostaglandin E2 (PGE2). On day 9 matured DCs were collected, washed and resuspended in sterile saline solution. A 10×10^6^ DCs dose was immediately administered to the pt, while the rest of obtained cells was cryopreserved in 90% autologous plasma and 10% DMSO solution until further use. All reagents used are listed in Supplementary Table 1.

***Quality control of DC vaccine***

***Safety test***

Endotoxin (≤ 0.5 EU/mL), germ-free and mycoplasma free tests were performed in accordance with European Pharmacopoeia guidelines.^46^

***DC Immunophenotype and Co-flow assay***

DC phenotype was determined by flow cytometry using a BD FACSCanto flow cytometer. The following monoclonal antibodies (Abs) with related isotype controls were used: anti-CD80, anti-CD86, anti-HLA-DR, and anti-CD83.

The potency of each DC batch was assessed by applying the Co-flow DC Assay, already described in detail.^16^ Briefly, healthy donor-derived CD3+ lymphocytes were labeled with PKH67 Green Fluorescent Cell Linker Midi Kit and co-cultured in triplicate with live allogeneic DCs at different conditions: 1×10^4^ live DCs and 1×10^5^ live T cells (background), the same number of cells were seeded with 0.005 µg/mL OKT-3 (COSTIM). Control conditions are included to confirm the assay evaluability. At the end of the 68 h of co-culture at 37° C in a humidified atmosphere, cells were analyzed by flow cytometry. The potency was calculated by subtracting the average T cells proliferation value of the background to the average T cells proliferation value of the COSTIM condition. Data analysis on T cell proliferation was performed using the Modfit LT 4.1 Software. All reagents, kits and softwares used are listed in Supplementary Table 1.

***DTH skin testing***

The DTH test consisted in the execution of subcutaneous injections of scalar concentrations (5, 10, 20, 50 and 100 μg) of KLH or ATH with the aim of verifying the different degrees of reactivity and sensitization to the antigens tested. An injection of saline solution alone served as a negative control. DTH tests were performed on arm A pts and every 12 weeks starting from the first vaccine. All reagents used are listed in Supplementary Table 1.

***INF-y ELISPOT assay***

Immunological efficacy of the treatment was evaluated by the IFN-γ ELISPOT assay kit in compliance with the MIATA guidelines, in order to observe IFN-γ secreting circulating effector cells activated by selected TAAs. Specifically, the peptides analyzed in this assay were Melan-A, PMEL, Tyrosinase, MAGE-A3, NY-ESO1 and survivin. Every peptide pool and CEF extended control peptides were reconstituted with dimethyl sulfoxide (DMSO) and diluted with AIM-V medium to the use concentration of 2 μg/mL.

On day 1, 20x10^6^ PBMC samples were thawed in RPMI 1640 medium added with Fetal Bovine Serum (FBS) 10% and DNase 1000 U, centrifuged, resuspended in AIM-V medium at the concentration of 2x10^6^ live PBMCs/mL and rested overnight (o/n) at 37°C in a humidified atmosphere. On day 2, the samples were tested with Annexin V/Propidium Iodide (Ann-V/PI) assay to accurately determine cell viability before the cell seeding. Moreover, a 96-well plate equipped with PVDF membranes were incubated with 15 μl of ethanol 70% for 2 minutes at room temperature (RT). Then, 50 μl of the coating anti-IFN-γ Ab (1:100) was added to each well and incubated o/n in the dark at 4°C. On day 2, the blocking solution was added to each well and incubated in the dark at 37°C for 1 h. The pt PBMC samples (2.22x10^6^ Ann-V-/PI- cells/mL) were seeded in triplicate (1,66x10^5^ live cells for 3 wells) with medium only (negative control) or stimulated with the CEF peptide pool (positive control) or selected peptides, and incubated o/n in the dark at 37°C. Each plate was set up including a biological replicate represented by an healthy donor (HD), with known reactivity against CEF peptide pool and 50 ng/mL of phorbol 12-myristate 13-acetate (PMA) as mitogenic positive control. The background condition, to further ensure the success of the assay, was evaluated with a solution of DMSO and AIM-V. On day 3, 100 μl of the secondary biotinylated Ab (1:100) was added to each well and incubated in the dark at 37°C for 1 h. Then, HRP-conjugated streptavidin was added to each well and incubated in the dark at 37°C for 1 h. Finally, the substrate was diluted in ethanol 30%, added to each well and incubated for 25 minutes at RT. Every step was followed by a careful washing stage with PBS 1X. PVDF membranes were dried in the dark at RT, and on day 6 evaluated using the ELISpot Reader and software. Numerous preventive determinations performed on different HDs, tested on positive and negative controls, allowed to apply standard spot reading parameters and to exclude artifacts and faint small background spots. Thus, the software could detect the secretion of IFN-γ as a spot of red substrate precipitated by each activated effector cell. The mean value of spot-forming cells (SFCs) detected in negative control wells was subtracted from the mean value of SFCs observed following peptide stimulation with the aim of obtaining a value not conditioned by a physiological immune response. All reagents, kits and softwares used are listed in Supplementary Table 1.

***Immunohistochemistry***

Evaluation of melanoma TAAs (PMEL, Melan-A, tyrosinase, NY-ESO1, survivin), immune infiltrate markers (CD8/CD163, CD68/FOXP3) and PD-L1 expression was performed by IHC staining on 4 μm formalin-fixed paraffin-embedded (FFPE) tissue sections taken from pre-treatment tumor biopsies. For pt #006 was also possible to obtain and analyze the post-treatment tumor biopsy. Detection was carried out using the UltraView Universal AP Red Detec Kit (for TAAs detection to avoid melanin interference) and OptiView DAB Detection Kit on the Ventana Benchmark ULTRA automatized coloring system as previously described.^47^ High-resolution whole slide images (WSI) (40× magnifications) of stained slides were acquired using the Aperio CS2 slide scanner. Semi-quantitative analysis of TAAs expression was performed to determine the percentage of positive cells. Briefly, the staining intensity was scored from 0 to 3 on five representative fields. PD-L1 TPS was calculated as previously reported.[^18^](https://sciwheel.com/work/citation?ids=16808414&pre=&suf=&sa=0)^,48^ All the analyses were carried out by a senior pathologist blinded to pt’s clinicopathological and survival information. All reagents, instruments and softwares used are listed in Supplementary Table 1.

***Immune cell immunophenotype by polychromatic flow cytometry***

A multicolor flow cytometry-based approach was used to assess frequency and phenotype of the major circulating myeloid and lymphoid subpopulations, before (PRE), during (VAX4) and after treatment (EOT, 1 month after the last vaccine).

PBMCs were thawed in RPMI 1640 supplemented with 10% FBS and 1000 U DNase, live and dead cells were discriminated by trypan blue exclusion method and 0.5x10^6^ or 1x10^6^ (for the enumeration of regulatory T cells (Tregs)) of live cells were stained for each panel. Pt’s samples taken at different time points were tested within the same experimental session, in accordance with “minimal information about T cell (MIATA) assays” guidelines, to improve the data quality level of flow cytometry assay. Live and dead cells were discriminated with LIVE/DEAD amine-reactive dyes and non-viable cells excluded during analysis and samples showing viability less than 60% were not further processed. For T regs enumeration, cells were washed, fixed, and permeabilized using Foxp3/Transcription Factor Staining Buffer Set following manufacturer's instructions.

Data was acquired on Attune NxT flow cytometer and analyzed using FlowJo V.10 software. In order to give statistical significance to poorly expressed or even rare cell populations, up to 200,000 events gated on single and live cells were acquired for each sample. Abnormal or manifestly artifact samples were excluded from analysis (e.g., light scatter or any fluorescence abnormal profile). All reagents, instruments and softwares used are listed in Supplementary Table 1.

***Multiplex cytokine immunoassay/Circulating Cytokines evaluation***

Circulating cytokine levels were tested in pt’s serum samples collected before, during and after treatment by using the flow cytometry bead-based array with a MACSPlex Cytokine kit for the simultaneous detection of human GM-CSF, IFN-α, IFN-γ, IL-2, IL-4, IL-5, IL-6, IL-9, IL-10, IL-12p70, IL-17A and TNF-α. Samples were evaluated using a MACSQuant Analyzer 10 and the Express Mode option of the MACSQuantify 2.8 software. In addition, the concentration of human IL-8 was measured with the Ella Automated Immunoassay System following the manufacturer's instructions. All reagents, instruments and softwares used are listed in Supplementary Table 1.

***Statistical analysis***

This study was an open-label randomized phase II trial (1:1 allocation ratio) in resected stage III/IV melanoma patients.^15^ The randomization lists were computer-generated using permutated blocks of varying sizes stratified by stage (III and IV M1a-b and IVM1c) and time from primitive tumor to first metastasis (≤2 years versus >2 years) by the Unit of Biostatistics and Clinical Trials of IRCCS-IRST. The sequences were concealed from the physicians. Patients were enrolled by the physicians and assigned to intervention by the personnel of the Unit of Biostatistics and Clinical Trials of IRCCS-IRST on the basis of the randomization list previous generated. An electronic case report form of the study was created and managed by the Unit of Biostatistics and Clinical Trials of IRCCS-IRST.

Primary endpoint was RFS and secondary endpoints were OS, *in vivo* and *in vitro* immune response evaluation, toxicity, prognostic and predictive markers of response. Based on previous studies, a median relapse-free survival of 7.0 months was assumed for the standard group. With a two-sided tailed alpha of 0.10 and power of 80%, assuming a median RFS of 11.7 months in the experimental arm (hazard ratio 0.60), it was planned to recruit 60 pts per arm over a period of 24 months and to follow-up for 12 months. In the context of data monitoring board activities, an interim analysis for futility, according to the Bayesian approach, was planned at 18 months in order to check for safety. During the enrolment phase however, several drugs were approved as adjuvant therapy, resulting in the premature stopping of pts accrual in the trial because follow up arm was no longer ethical. RFS was defined as the time elapsed between the date of randomization and the date of first relapse or the date of death from any cause or the date of the last restaging in non-relapsed (NR) pts. OS was defined as the time elapsed between the date of randomization and the date of death from any cause or the last date on which the patient was known to be alive. RFS and OS were analyzed using the Kaplan-Meier method and compared using a log-rank test. Median RFS and OS were reported, as were the corresponding 95% confidence intervals (CIs). The median follow-up time was computed by the reverse Kaplan-Meier approach and reported together with 95% CIs. Hazard ratios and their 95% CIs were estimated using Cox proportional hazard regression model. The paired Student t-test or the Wilcoxon signed rank test was used, as appropriate, to compare baseline and EOT values. An analysis of variance (ANOVA) for repeated measures was used to explore the potential changes over time of biomarkers. No adjustment for potential confounding factors was performed. Since the study did not reach the required sample size (n=120), all the analyses presented in this paper are intended to be hypothesis-generating, with a significance level set at *p*<0.05, without adjustments for multiplicity. All of the analyses were carried out with SAS software.
