# Supplementary material for: Adjuvant dendritic cell-based immunotherapy in melanoma: insights into immune cell dynamics and clinical evidence from a phase II trial
Source: J Transl Med. 2025 Apr 18;23:455. doi: 10.1186/s12967-025-06403-8 (PMC12007200; doi:10.1186/s12967-025-06403-8)
Supplement: Supplementary file 2 — Additional file 2. Details of reagents, kits, softwares and instruments used in the study [file 12967_2025_6403_MOESM2_ESM.pdf]

Details of reagents, kits, softwares and instruments

Antibodies	RRID	Panel	Manufacturer	Label	Clone	Ctalog #	Usage	Technique
CD3	AB_1834395	1; mono/MDSC & MDSC/CD84; T activation/diffe	ThermoFisher Scientific, Massachusetts, U	PerCP-eFluor710	OKT3	46-0037-42	2,5 uL	Multiparametric FACS
CD3	AB_1272042	2; CD4 Th & Tregs	ThermoFisher Scientific, Massachusetts, U	APC-eFluor 780	UCHT1	47-0038-42	5 uL	Multiparametric FACS
CD4	AB_2726691	2; CD4 Th & Tregs4; T activation/differentiation/ex	Miltenyi Biotec, Bergisch Gladbach, Germa	VioBlue	REA623	130-114-534	1 uL	Multiparametric FACS
CD8	AB_2659239	1; mono/MDSC & MDSC/CD8	Miltenyi Biotec, Bergisch Gladbach, Germa	VioBlue	REA734	130-110-683	2 uL	Multiparametric FACS
CD8	AB_11218688	4; T activation/differentiation/exhaustion	eBioscience, San Diego, CA, USA	Alexa Flour 700	RPA-T8	56-0088-42	2,5 uL	Multiparametric FACS
CD11b	AB_2654675	1; mono/MDSC & MDSC/CD8	Miltenyi Biotec, Bergisch Gladbach, Germa	APC-Vio 770	REA713	130-110-556	2 uL	Multiparametric FACS
CD14	AB_2655057	1; mono/MDSC & MDSC/CD8	Miltenyi Biotec, Bergisch Gladbach, Germa	VioGreen	REA599	130-110-525	2 uL	Multiparametric FACS
CD16	AB_2726149	1; mono/MDSC & MDSC/CD8	Miltenyi Biotec, Bergisch Gladbach, Germa	APC	REA423	130-113-389	1 uL	Multiparametric FACS
CD19	AB_10671142	1; mono/MDSC & MDSC/CD8	ThermoFisher Scientific, Massachusetts, U	PerCP-eFluor710	SJ25C1	46-0198-42	2,5 uL	Multiparametric FACS
CD25	AB_2727131	2; CD4 Th & Tregs	Miltenyi Biotec, Bergisch Gladbach, Germa	PE Vio 615	REA945	130-115-631	2 uL	Multiparametric FACS
CD27	AB_10717249	4; T activation/differentiation/exhaustion	eBioscience, San Diego, CA, USA	PE-CY5.5	323	15-0279-42	5 uL	Multiparametric FACS
CD28	AB_2762537	4; T activation/differentiation/exhaustion	eBioscience, San Diego, CA, USA	Super Bright 600	CD28.2	63-0289-41	5 uL	Multiparametric FACS
CD33	AB_2657565	1; mono/MDSC & MDSC/CD8	Miltenyi Biotec, Bergisch Gladbach, Germa	PE-Vio 615	REA775	130-111-026	2 uL	Multiparametric FACS
CD38	AB_2733228	4; T activation/differentiation/exhaustion	Miltenyi Biotec, Bergisch Gladbach, Germa	PE-Vio 770	REA572	130-113-432	2 uL	Multiparametric FACS
CD45RA	AB_2662460	2; CD4 Th & Tregs	eBioscience, San Diego, CA, USA	Super Bright 702	HI100	67-0458-42	5 uL	Multiparametric FACS
CD45RA	AB_2726133	4; T activation/differentiation/exhaustion	Miltenyi Biotec, Bergisch Gladbach, Germa	APC-Vio770	REA562	130-113-363	1 uL	Multiparametric FACS
CD56	AB_2637487	1; mono/MDSC & MDSC/CD8	eBioscience, San Diego, CA, USA	PerCP-eFluor710	TULY56	46-0566-42	5 uL	Multiparametric FACS
CD57	AB_2737625	4; T activation/differentiation/exhaustion	BD Pharmingen, San Diego, CA, USA	PE CF594	NK-1	562488	0,2 uL	Multiparametric FACS
CD66b	AB_2811406	1; mono/MDSC & MDSC/CD8	Miltenyi Biotec, Bergisch Gladbach, Germa	PE	REA306	130-122-922	2 uL	Multiparametric FACS
CD80	AB_2802016	DC Immunophenotype	Miltenyi Biotec, Bergisch Gladbach, Germa	PE	REA661	130-123-253	5 uL	Multiparametric FACS
CD83	AB_3674840	DC Immunophenotype	Beckman Coulter, Milan, Italy	FITC	HB15a	IM2410U	4 uL	Multiparametric FACS
CD86	AB_396651	DC Immunophenotype	BD Pharmingen, San Diego, CA, USA	FITC	2331 (FUN-1)	557343	5 uL	Multiparametric FACS
CD95	AB_2738021	4; T activation/differentiation/exhaustion	BD Pharmingen, San Diego, CA, USA	BV711	DX2	563132	5 uL	Multiparametric FACS
CD183 (CXCR3)	AB_2572588	2; CD4 Th & Tregs	ThermoFisher Scientific, Massachusetts, U	PE	CEW33D	12-1839-41	5 uL	Multiparametric FACS
CD194 (CCR4)	AB_2751493	2; CD4 Th & Tregs	Miltenyi Biotec, Bergisch Gladbach, Germa	PE-Vio 770	REA279	130-118-359	2 uL	Multiparametric FACS
CD196 (CCR6)	AB_10597900	2; CD4 Th & Tregs	ThermoFisher Scientific, Massachusetts, U	PerCP-eFluor™ 710	R6H1	46-1969-42	2,5 uL	Multiparametric FACS
CD197 (CCR7)	AB_2784045	4; T activation/differentiation/exhaustion	Miltenyi Biotec, Bergisch Gladbach, Germa	PE	REA108	130-120-463	2 uL	Multiparametric FACS
CD274 (PDL1)	AB_1907368	1; mono/MDSC & MDSC/CD8	ThermoFisher Scientific, Massachusetts, U	PE-CY7	MIH1	25-5983-42	5 uL	Multiparametric FACS
CD279 (PD1)	AB_2728018	2; CD4 Th & Tregs4; T activation/differentiation/ex	Miltenyi Biotec, Bergisch Gladbach, Germa	VioBright FITC	PD 1.3 1.3	130-117-681	1 uL	Multiparametric FACS
FOXP3	AB_1603280	2; CD4 Th & Tregs	ThermoFisher Scientific, Massachusetts, U	APC	PCH101	17-4776-42	5 uL	Multiparametric FACS
HLA-DR	AB_398674	4; T activation/differentiation/exhaustion	BD Pharmingen, San Diego, CA, USA	APC	G46-6	559866	2,5 uL	Multiparametric FACS
HLA-DR	AB_2652156	1; mono/MDSC & MDSC/CD8	Miltenyi Biotec, Bergisch Gladbach, Germa	FITC	REA805	130-111-788	1 uL	Multiparametric FACS
HLA-DR	AB_395943	DC Immunophenotype	BD Pharmingen, San Diego, CA, USA	PE	TU36	555561	5 uL	Multiparametric FACS
ki67	AB_2637481	2; CD4 Th & Tregs	ThermoFisher Scientific, Massachusetts, U	eFluor 506	SolA15	69-5698-80	2,5 uL	Multiparametric FACS
LIVE/DEAD®	N.A.	4; T activation/differentiation/exhaustion	ThermoFisher Scientific, Massachusetts, U	Fixable Aqua	/	L34957	1 uL	Multiparametric FACS
LIVE/DEAD®	N.A.	1; mono/MDSC & MDSC/CD82; CD4 Th & Tregs	ThermoFisher Scientific, Massachusetts, U	Fixable Yellow	/	L34959	1 uL	Multiparametric FACS

Antibodies	RRID	Manufacturer	Isotype/Host	Cromogen	Clone	Ctalog #	Usage	Technique
CD163	AB_2920861	Leica Biosystems, Nußloch, Germany	IgG1/Mouse monoclonal	AP RED (double staining	10D6	CD163-L-CE	1:50	IHC
CD8	AB_2335985	Roche, Basilea, Svitzerland	Rabbit Monoclonal Antibody	DAB (double staining)	SP57	790-4460	Prediluited	IHC
CD68	AB_306119	Abcam, Cambridge, UK	IgG3/Mouse monoclonal	AP RED (double staining	PG-M1	ab783	1:100	IHC
FOXP3	AB_2537884	ThermoFisher Scientific, Massachusetts, USA	IgG1/Rabbit monoclonal	DAB (double staining)	SP97	MA5-16365	1:100	IHC
Melan-A	AB_2335691	Dako by Agilent Technologies, Santa Clara, USA	IgG1k/Mouse monoclonal	AP RED	A103	M7196	1:15	IHC
NY-ESO1	AB_784921	Santa Cruz Biotechnologies, Dallas, Texas, USA	IgG1k/Mouse monoclonal	AP RED	E978	SC53869	1:25	IHC
PD-L1	AB_3674898	Roche, Basilea, Svitzerland	Recombinant rabbit monoclonal antibody	AP RED	SP263	741-4905	Prediluited	IHC
PMEL	AB_2335682	Dako by Agilent Technologies, Santa Clara, USA	IgG1k/Mouse monoclonal	AP RED	HMB45	M0634	Prediluited	IHC
Survivin	AB_2243439	Dako by Agilent Technologies, Santa Clara, USA	IgG2A kappa	AP RED	12C4	M3624	1:50	IHC
Tyrosinase	AB_2210866	Dako by Agilent Technologies, Santa Clara, USA	IgG2A kappa/Mouse monoclonal	AP RED	T311	M3623	1:15	IHC

Peptides	RRID	Manufacturer	Ctalog #	Usage	Assay
CEF (extended)	N.A.	JPT Technology, Berlin, Germany	PM-CEF-E-1	2 µg/ml	Elispot assay
Human IFN-γ antibody pair	N.A.	U-CyTech biosciences, Utrecht, The Netherlands	CT640-10	According to manufactur	Elispot assay
MAGE-A3	N.A.	JPT Technology, Berlin, Germany	PM-MAGEA3	2 µg/ml	Elispot assay
Melan-A	N.A.	JPT Technology, Berlin, Germany	PM-MelA	2 µg/ml	Elispot assay
NY-ESO1	N.A.	JPT Technology, Berlin, Germany	PM-NYE	2 µg/ml	Elispot assay
PMA	N.A.	Sigma Aldrich, St. Louis, MO, USA	P1585	50 ng/ml	Elispot assay
PMEL	N.A.	JPT Technology, Berlin, Germany	PM-GP100	2 µg/ml	Elispot assay
Survivin	N.A.	JPT Technology, Berlin, Germany	PM-Survivin	2 µg/ml	Elispot assay
Tyrosinase	N.A.	JPT Technology, Berlin, Germany	PM-Tyr	2 µg/ml	Elispot assay

Other Reagents/Kits					
**Name**	**RRID**	**Vendor**	**Catalog number**	**Usage**	**Technique/ assay**
AIM-V Medium	N.A.	Gibco, Thermo Fisher Scientific, Waltham, MA, US	12055091	N.A.	Elispot assay
Annexin V-FITC Apoptosis Kit	AB_2575600	Invitrogen, Carlsbad, CA, USA	BMS500FI-300	According to manufacture	Multiparametric Flow Cytofluorimetry
CellGenix® GMP DC Medium	N.A.	Sartorius CellGenix GmbH, Freiburg, Germany	20801-0500	According to manufacture	DC preparation
DMSO	N.A.	Mylan, Milan, Italy	674570-178-50	N.A.	Elispot assay
DNase I (RNase free)	N.A.	Ambion by Life tecnologies, Carlsbad, California, U	AM2222	2 U/µL	Cellular Biology
IFN-γ ELISPOT assay kit	AB_418361	U-CyTech biosciences, Utrecht, The Netherlands	CT230	According to manufacture	Elispot assay
FBS	N.A.	EuroClone, Milan, Italy	ECS5000D	N.A.	Cellular Biology
Foxp3/Transcription Factor Staining Buffer	N.A.	eBioscience, San Diego, CA, USA	00-5523-00	According to manufacture	Multiparametric Flow Cytofluorimetry
IL-8 Cartridge	N.A.	Biotechne, Milan, Italy	SPCKB-PS-000230	According to manufacture	Cytokine immunoassay
Immucothel (KLH)	N.A.	Biosyn, Carlsbad, United States	RNG18971	50 mg/mL	Cellular Biology
Lifecodes HLA-A, B, C, DRB1, DQA, DQB	N.A.	Immucor Transplant Diagnostics Inc	628913, 628917, 628921, 628925, 628930	According to manufacture	Molecular Biology
Lymphocyte separation media	N.A.	Biowest, Riverside, MO, USA	L-0560	According to manufacture	Cellular Biology
MACSPlex Cytokine kit	N.A.	Miltenyi Biotec, Bergisch Gladbach, Germany	130-099-169	According to manufacture	Multiparametric Flow Cytofluorimetry
Maxwell® RSC Whole Blood DNA Kit	N.A.	Promega Corporation, Madison, WI	AS1520	N.A.	Cellular Biology
OKT-3	AB_1951245	Abcam, Cambridge, UK	ab86883	0.005 µg/mL	DC potency
PGE2	N.A.	Cayman Chemical, Ann Arbor, MI, USA	CAY-14010-1	1 µg/mL	DC preparation
PKH67 Green Fluorescent Cell Linker Midi	N.A.	Sigma Aldrich, St. Louis, MO, USA	MIDI67	According to manufacture	DC potency
PVDF 96-well plate	N.A.	Merck Life Science, Milan, Italy	MSDVN6B50	N.A.	Elispot assay
rh IL-4	N.A.	Sartorius CellGenix GmbH, Freiburg, Germany	1003	1000 IU/mL	DC preparation
rh GM-CSF	N.A.	Sartorius CellGenix GmbH, Freiburg, Germany	1012	1000 IU/mL	DC preparation
rh IL-6	N.A.	Sartorius CellGenix GmbH, Freiburg, Germany	1004	2000 IU/mL	DC preparation
rh TNFα	N.A.	Sartorius CellGenix GmbH, Freiburg, Germany	1006	20 ng/mL	DC preparation
rh IL-1β	N.A.	Sartorius CellGenix GmbH, Freiburg, Germany	1011	20 ng/mL	DC preparation
RPMI 1640 Medium	N.A.	EuroClone, Milan, Italy	ECB9006L	N.A.	Cellular Biology
Trypan Blue	N.A.	Gibco by Life tecnologies, Carlsbad, California, US	15250061	N.A.	Cellular Biology
OptiView DAB Detection Kit	N.A.	Ventana-Roche, Oro Valley, Arizona, USA	6396500001	According to manufact	IHC
UltraView Universal AP Red Detection K	N.A.	Ventana-Roche, Oro Valley, Arizona, USA	5269814001	According to manufact	IHC

Instruments/Softwares			
**Name**	**RRID**	**Vendor**	**Technique/ assay**
Aperio CS2 slide scanner	SCR_025111	Leica Biosystems, Nußloch, Germany	IHC
Attune NxT flow cytometer	SCR_019590	Thermo Fisher, Waltham, Massachusetts, USA	Multiparametric Flow Cytofluorimetry
BD Scientific Canto II Flow Cytometer	SCR_018056	Becton Dickinson, Milan, Italy	Multiparametric Flow Cytofluorimetry
ELLA	N.A.	Biotechne, Milan, Italy	Cytokine immunoassay
FlowJo V.10	SCR_008520	Becton, Dickinson and Ashland, Oregon, USA	Multiparametric Flow Cytofluorimetry
Luminex Flow Analyser	SCR_018025	Luminex Corporation, Austin, TX USA	Molecular Biology
MACSQuant Analyzer 10	SCR_020268	Miltenyi Biotec, Bergisch Gladbach, Germany	Multiparametric Flow Cytofluorimetry
Maxwell® RSC	N.A.	Promega Corporation, Madison, WI	Cellular Biology
Modfit LT 4.1 Software	SCR_016106	Verity Software House, Topsham, ME, USA	Multiparametric Flow Cytofluorimetry
MACSQuantify 2.8	SCR_020943	Miltenyi Biotec, Bergisch Gladbach, Germany	Multiparametric Flow Cytofluorimetry
NanoDrop™ 3300	SCR_015804	ThermoFisher Scientific, Massachusetts, USA	Molecular Biology
SAS v 9.4	SCR_008567	SAS Inst, Cary, NC, USA	Statistical Analysis
Simple Plex 4.1	N.A.	Biotechne, Milan, Italy	Cytokine immunoassay
BenchMark ULTRA	SCR_025506	Ventana-Roche, Oro Valley, Arizona, USA	IHC
