# Supplementary figures and images for: Adjuvant dendritic cell-based immunotherapy in melanoma: insights into immune cell dynamics and clinical evidence from a phase II trial
Source: J Transl Med. 2025 Apr 18;23:455. doi: 10.1186/s12967-025-06403-8 (PMC12007200; doi:10.1186/s12967-025-06403-8)

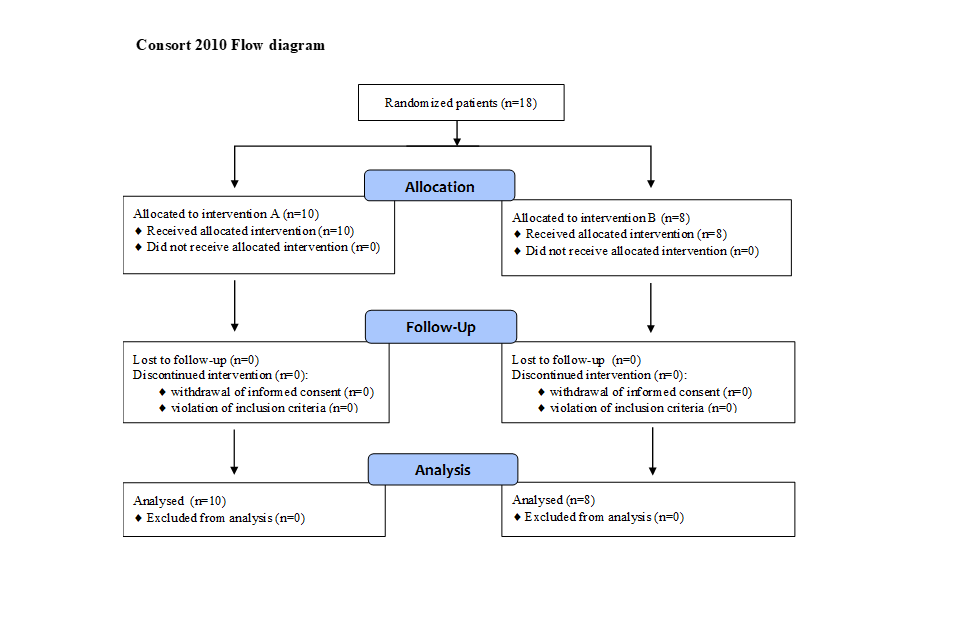

Supplement: Supplementary file 3 — Additional file 3. CONSORT Flow diagram of the clinical trial [file 12967_2025_6403_MOESM3_ESM.tif]

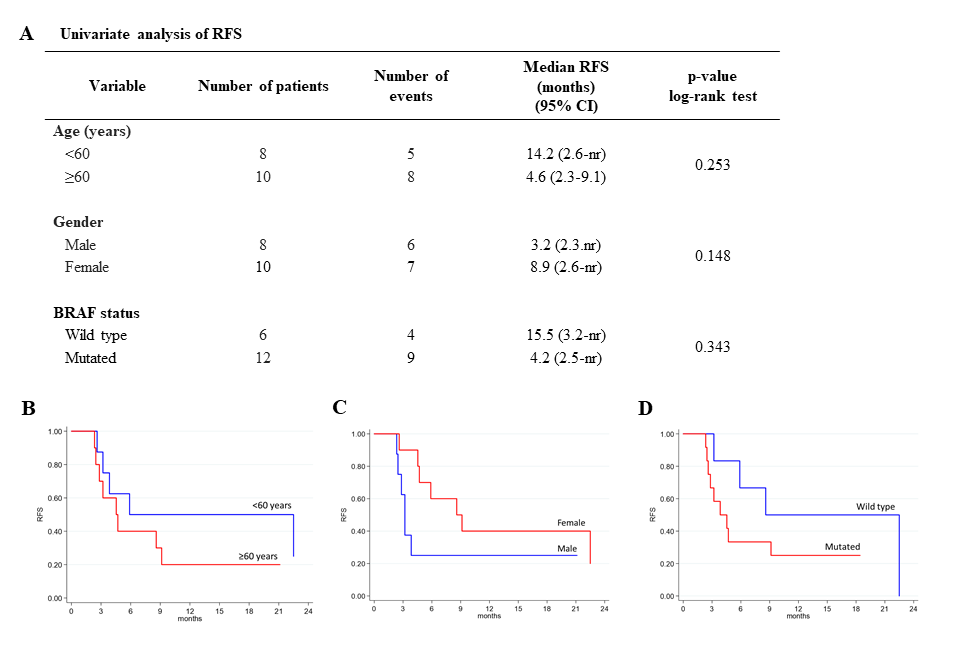

Supplement: Supplementary file 4 — Additional file 4. The table shows the univariate analysis of RFS of the entire pts cohort for age, gender and Braf gene status.Kaplan-Meier curve of the univariate analysis of RFS by age.Kaplan-Meier curve of the univariate analysis of RFS by gender.Kaplan-Meier curve of the univariate analysis of RFS by Braf gene status [file 12967_2025_6403_MOESM4_ESM.tif]
