# Supplementary material for: Adjuvant dendritic cell-based immunotherapy in melanoma: insights into immune cell dynamics and clinical evidence from a phase II trial
Source: J Transl Med. 2025 Apr 18;23:455. doi: 10.1186/s12967-025-06403-8 (PMC12007200; doi:10.1186/s12967-025-06403-8)
Supplement: Supplementary file 5 — Additional file 5. Number of patients with at least 1 cycle of treatment with reported adverse events [file 12967_2025_6403_MOESM5_ESM.pdf]

**Number of patients with at least 1 cycle of treatment with reported AEs.**

| | Arm A: Vaccine(n=10) N of patients (%) | | | | Arm B: Observation (n=8) N of patients (%) | | | |
|---|---|---|---|---|---|---|---|---|
| | **G1** | **G2** | **G3** | **G4** | **G1** | **G2** | **G3** | **G4** |
| Fever | 3 | 2 | 1 | 0 | 0 | 0 | 0 | 0 |
| Vomiting | 1 | 0 | 1 | 0 | 0 | 0 | 0 | 0 |
| Nausea | 0 | 1 | 1 | 0 | 0 | 0 | 0 | 0 |
| Diarrhea | 1 | 0 | 0 | 0 | 0 | 0 | 0 | 0 |
| Asthenia/Fatigue | 1 | 2 | 1 | 0 | 0 | 0 | 0 | 0 |
| Pruritus | 2 | 0 | 0 | 0 | 0 | 0 | 0 | 0 |
| Constipation | 0 | 0 | 1 | 0 | 0 | 0 | 0 | 0 |
| Respiratory system | 1 | 1 | 0 | 0 | 0 | 0 | 0 | 0 |
| Injection site reaction | 3 | 0 | 0 | 0 | 0 | 0 | 0 | 0 |
| Local reaction at vaccine injection site | 1 | 0 | 0 | 0 | 0 | 0 | 0 | 0 |
| Vaccination site reaction | 0 | 0 | 1 | 0 | 0 | 0 | 0 | 0 |
| Articular pain | 1 | 0 | 0 | 0 | 0 | 0 | 0 | 0 |
| Epigastric pain | 0 | 3 | 0 | 0 | 0 | 0 | 0 | 0 |
| Reflux | 0 | 1 | 0 | 0 | 0 | 0 | 0 | 0 |
| Vertigo | 1 | 0 | 0 | 0 | 0 | 0 | 0 | 0 |
| Dysgeusia | 0 | 1 | 0 | 0 | 0 | 0 | 0 | 0 |
| Dizziness | 0 | 0 | 0 | 0 | 1 | 0 | 0 | 0 |
| Hypertension | 0 | 0 | 0 | 0 | 1 | 0 | 0 | 0 |
| Symptomatic brain metastasis | 0 | 0 | 0 | 0 | 0 | 0 | 1 | 0 |
